# Iron Status, Erythropoietin, and Cancer Incidence in the General Population

**DOI:** 10.1111/eci.70218

**Published:** 2026-05-08

**Authors:** Siem J. van Alfen, Pien Rawee, Li Luo, Ilja M. Nolte, Bert van der Vegt, Jenny E. Kootstra‐Ros, Stephan J. L. Bakker, Ron T. Gansevoort, Thera P. Links, Wouter T. Zandee, Michele F. Eisenga

**Affiliations:** ^1^ Division of Endocrinology, Department of Internal Medicine, University Medical Center Groningen University of Groningen Groningen the Netherlands; ^2^ Division of Nephrology, Department of Internal Medicine, University Medical Center Groningen University of Groningen Groningen the Netherlands; ^3^ Department of Epidemiology, University Medical Center Groningen University of Groningen Groningen the Netherlands; ^4^ Department of Pathology, University Medical Center Groningen University of Groningen Groningen the Netherlands; ^5^ Department of Laboratory Medicine, University Medical Center Groningen University of Groningen Groningen the Netherlands

**Keywords:** cancer risk, EPO levels, general population, iron status, site‐specific cancer

## Abstract

**Background:**

Iron is essential for cellular function and cancer growth. While iron imbalance has been implicated in cancer development, epidemiological evidence remains inconsistent. Erythropoietin (EPO) may influence tumour progression. We aimed to investigate the associations between iron status, EPO levels, and cancer incidence in the general population.

**Method:**

Data were obtained from 6109 participants (mean age 52 ± 12 years; 49% male) in the Prevention of Renal and Vascular End‐stage Disease (PREVEND) cohort. Iron biomarkers, including ferritin, transferrin saturation, soluble transferrin receptor (sTfR), hepcidin and EPO levels, were measured at baseline.

**Results:**

Over a median 18.5 year follow‐up, 1090 participants developed cancer. Multivariable Cox regression revealed that higher EPO (HR 1.26; 95% CI 1.07–1.47; *p* = 0.005) was associated with increased overall cancer risk, while elevated hepcidin levels were associated with a lower risk (HR 0.88; 95% CI 0.80–0.96; *p* = 0.006). Higher sTfR (HR 1.35; 95% CI 1.01–1.80; *p =* 0.043) was suggestive for an increased risk of overall cancer. After excluding early diagnoses, the increased risk associated with higher EPO levels and decreased risk associated with higher hepcidin levels remained significant. Lower transferrin saturation was associated with increased haematological cancer risk, while higher hepcidin was associated with reduced gastrointestinal cancer risk, especially in women and those with BMI < 25 kg/m^2^. In non‐smokers, higher sTfR and EPO were associated with increased overall and kidney cancer.

**Conclusion:**

These findings underscore the putative roles of iron metabolism and EPO in cancer, with consistently decreased risks associated with elevated hepcidin levels, particularly among women and individuals with lower BMI.

## Graphical abstract

This study investigated the association between iron parameters and EPO and the development of overall and site‐specific cancer in a large cohort with a median follow‐up of 18.5 years (*n* = 6109; 1090 de novo malignancies). In multivariable cox proportional hazard analysis, we found that increased EPO, and decreased hepcidin concentrations are associated with the development of overall cancer, and increased sTfR was suggestive of an association with the development of overall cancer with smoking‐, sex‐ and BMI specific associations.

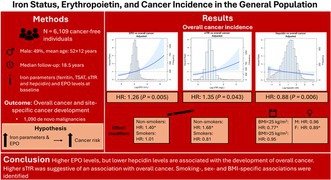

## 1 Introduction

Iron is an essential micronutrient involved in oxygen transport, DNA synthesis and energy production [1]. Iron is pivotal for red blood cell formation and supports key functions in brain and cardiovascular health [2]. Disordered iron homeostasis has been implicated in numerous diseases, ranging from heart failure to neurological disorders [3, 4].

Iron metabolism is also frequently altered in cancer. Elevated intracellular iron increases the labile iron pool, promoting reactive oxygen species formation, which can damage DNA and foster tumour growth [5]. Conversely, iron deficiency may impair immune surveillance and DNA repair, potentially increasing cancer risk [5]. A large Taiwanese study reported higher rates of pancreatic, liver, kidney and bladder cancer in patients with iron deficiency anaemia [6]. In addition, cancer‐related anaemia, commonly involving functional iron deficiency due to inflammation and tumour‐driven changes, can impair red blood cell production [7]. Inflammatory cytokines, particularly IL‐6, are able to induce hepcidin production. Hepcidin promotes the degradation of ferroportin, the only known cellular iron exporter, further contributing to cancer‐related anaemia [5].

Although experimental studies show excess iron may promote cancer, human data remain inconsistent [8, 9, 10, 11]. Notably, previous population‐based studies have not considered serum erythropoietin (EPO) levels when examining iron status and cancer risk. Iron status and EPO are closely linked, and elevated EPO has been observed in certain cancers, while exogenous EPO use has been associated with increased tumour risk [11]. Importantly, previous studies failed to account for reverse causation, as cancer‐related metabolic changes may influence iron markers before diagnosis, potentially biasing associations.

Here, we evaluate the association between a comprehensive panel of systemic iron markers and cancer incidence, while accounting for EPO levels, in a large, population‐based cohort.

## 2 Methods

### 2.1 Study Design and Population

For this prospective, observational study, we used the Prevention of Renal and Vascular End‐stage Disease (PREVEND) study, the details of which have been described elsewhere [12]. For the current analyses, we used data from the second survey, which took place between 2001 and 2003 (*n* = 6894), since for this visit, blood samples were also available.

### 2.2 Data Collection

The procedures for each examination in the PREVEND study have been described in detail previously [12]. Detailed descriptions of laboratory measurements including iron status parameters and EPO can be found in the Supplementary Methods (M1).

### 2.3 Definition of Cancer Outcomes

The primary outcome was the incidence of overall cancer. Secondary outcomes were the incidence of site‐specific cancers. The site‐specific cancers with an event rate exceeding 0.5% in our study population are reported [13]. Non‐melanoma skin cancer was excluded from the definition of overall cancer due to its high prevalence and benign prognosis [14]. Subjects with a cancer diagnosis before baseline were excluded from the analyses of the corresponding cancer type and for overall cancer incidence. For cancer incidence, data were retrieved via linkage to the Dutch Nationwide Pathology Databank (Palga). Palga data were obtained from 1971 to 2015 [15]. In addition to Palga data, we used a self‐reported questionnaire to exclude subjects with cancer before baseline. In case of multiple cancer diagnoses during follow‐up, the earliest cancer diagnosis after baseline was used to index overall cancer. Subjects were censored at the end of follow‐up (31 December 2015) or at the date of non‐cancer death, whichever occurred first. Individuals with a cancer diagnosis prior to the moment of blood drawing were excluded. Finally, we took into account individuals who had both data available on iron status parameters and EPO levels (at least one parameter) and cancer follow‐up resulting in a final cohort of 6109 community‐dwelling individuals.

### 2.4 Statistics

Data were analysed using R version 3.2.3 (Vienna, Austria). Baseline characteristics are described as means with standard deviation for normally distributed variables, medians with interquartile range for skewed variables, or percentages for categorical variables. Differences in baseline characteristics across tertiles of iron status parameters and EPO levels were tested using linear regression for normally distributed variables, Kruskal–Wallis test for skewed variables and chi‐squared test for categorical variables.

To study the associations of iron status parameters and EPO levels, we performed Cox proportional hazard regression analyses to estimate hazard ratios with 95% confidence intervals. Proportionality in Cox regression analyses was checked using Schoenfeld residuals plots and no violation was detected. Statistical significance was defined as *p* < 0.05 for overall cancer and *p* < 0.00125 for site‐specific cancers, with the latter threshold determined using a Bonferroni correction for multiple testing across 40 Cox regression models. Furthermore, we generated Kaplan–Meier curves to show the associations of tertiles of iron status parameters and EPO with the incidence of overall cancer. A log‐rank test for trend was used to compare cancer incidence across tertiles. Multivariable analysis was adjusted for socio‐demographic factors (age, sex, race, education (low, middle, high), smoking (never, former, present), and alcohol consumption (never, moderate, severe)), laboratory parameters (body mass index (BMI), estimated glomerular filtration rate (eGFR), urinary albumin excretion (UAE), hs‐CRP level, total cholesterol, and total serum protein), and clinical comorbidities (hypertension, antihypertensives use, diabetes type 2, antidiabetic medication use and lipid lowering drugs use). Multivariable models were constructed in a stepwise fashion. Model 1 included age, sex and race. Model 2 further incorporated all additional socio‐demographic, laboratory and clinical covariates. Ferritin, sTfR, hepcidin and EPO were log‐transformed.

We performed several sensitivity analyses. First, to detect possible reverse causation, we did a 1‐year landmark analysis by excluding subjects with a cancer diagnosis < 1 year following blood sampling and remodelled the associations. Second, we used the Bonferroni correction to account for multiple testing for the site‐specific cancers (*α* = 0.00625 with 8 site‐specific cancers). Third, we explored potential effect modification by demographic and clinical subgroups: age (≤ 65 years vs. > 65 years), sex (male vs. female), smoking status (current non‐smoker vs. current smoker), BMI (≤ 25 kg/m^2^ vs. > 25 kg/m^2^) and eGFR (≤ 90 mL/min/1.73 m^2^ vs. > 90 mL/min/1.73 m^2^). Finally, we assessed whether commonly used cut‐offs in clinical practice, that is, ferritin < 30 μg/L for iron deficiency or TSAT > 45% for iron overload, were associated with the development of cancer. Overall, 4.1% of the data were missing and were imputed using regressive switching. Five datasets were multiply‐imputed, and results were pooled according to Rubin's rules [16]. All *p*‐values are two‐tailed and a *p* < 0.05 is considered statistically significant.

## 3 Results

### 3.1 Baseline Characteristics

We included 6109 community‐dwelling individuals (age 52 ± 12 years; 49% males; 96% Caucasian) with a median ferritin level of 96 (Interquartile range [IQR]: 47–171) μg/L, mean TSAT of 25.0% ± 9.5%, median sTfR 2.48 (IQR: 2.11–2.99) mg/L, median hepcidin 3.01 (IQR: 1.64–4.87) nM and median EPO level of 7.76 (IQR: 5.9–9.15) IU/L. Across tertiles of ferritin, the primary marker of iron homeostasis, we identified that individuals with higher ferritin levels were older, more likely to be male, more likely to be diabetic, less likely to smoke, consume more alcoholic beverages, more likely to have a higher BMI, and to have higher levels of hs‐CRP and UAE (Table 1A). Across tertiles of EPO levels, participants were older, more likely to be female, have a lower education and higher BMI levels. In addition, no alcohol use or currently smoking was associated with a higher EPO concentration (Table 1B). Additional baseline characteristics can be found in Tables 1A and 1B. Baseline characteristics across tertiles of the other iron status parameters, that is, TSAT, sTfR and hepcidin, can be found in Tables S1–S3.

**TABLE 1A:** Baseline characteristics of the community‐dwelling individuals across tertiles of ferritin levels.

		Ferritin tertiles (μg/L)			
		T1 < 61	T2: 61–139	T3 > 139	*p* for trend
Baseline characteristics					
Cancer incidence	18.8%	14.7%	18.6%	20.6%	< 0.001
Age, years	52.7 (11.9)	48.7 (11)	53.8 (11.7)	55.5 (11.9)	< 0.001
Male, %	49.2%	21.5%	52.2%	74%	< 0.001
Caucasian, %	96%	96.1%	96.6%	95.2%	0.174
High education, %	31%	35.5%	29.4%	28.1%	< 0.001
Current smoker, %	28.3%	28.9%	29.2%	26.8%	< 0.001
No alcoholic beverages, %	23.7%	29.5%	22.6%	19%	< 0.001
Clinical parameters					
BMI, kg/m^2^	26.7 (4.4)	25.6 (4.4)	26.6 (4.2)	27.9 (4.2)	< 0.001
SBP, mmHg	125.9 (18.7)	119.9 (17.2)	126.5 (18.8)	131.2 (18.4)	< 0.001
eGFR, mL/min/1.73 m^2^	94.2 [81.9–104.5]	97.5 [85.8–106.9]	92.7 [80.9–103.8]	92 [79.5–102]	< 0.001
UAE, mg/24 h	8.5 [6–15.1]	7.9 [5.7–12.8]	8.2 [6–14.6]	9.6 [6.6–18.8]	< 0.001
Hs‐CRP, mg/L	1.3 [0.6–3]	1 [0.5–2.5]	1.3 [0.6–2.9]	1.7 [0.8–3.5]	< 0.001
Serum protein, g/L	67.1 (4.1)	66.4 (4.1)	66.8 (4.1)	68 (4)	< 0.001
HDL, mg/dL	48.5 (12.1)	52.3 (11.9)	48.7 (12.1)	44.6 (11.22)	< 0.001
Total cholesterol, mmol/L	5.4 [4.7–6.1]	5.1 [4.5–5.8]	5.4 [4.8–6.1]	5.6 [4.9–6.3]	< 0.001
Comorbidities and medication use					
Hypertension, %	33.4%	21.5%	34.2%	44.4%	< 0.001
Use of antihypertensives, %	17.5%	10.9%	17.2%	24.2%	< 0.001
Use of lipid lowering drugs, %	9.5%	6.2%	9.9%	12.5%	< 0.001
Diabetic, %	5.9%	3.5%	5.2%	9.2%	< 0.001
Use of antidiabetic medication, %	1.2%	1%	1.4%	1.2%	< 0.001
Red blood cell and iron parameters					
Ferritin, μg/L	96 [47–171]				
EPO, IU/L	7.76 [5.9–9.15]	8.4 [6.2–11.4]	7.6 [5.9–10]	7.4 [5.6–9.5]	< 0.001
Hb, g/dL	14.1 (1.3)	13.4 (1.2)	14.3 (1.1)	14.7 (1.1)	< 0.001
MCV, fL	90.4 (4.6)	89.3 (5.2)	90.8 (4.1)	91.2 (4.4)	< 0.001
TSAT, %	25.1 (9.5)	21.7 (9.6)	25.5 (8.5)	28 (9.4)	< 0.001
Hepcidin, nM	3 [1.6–4.9]	1.2 [0.6–2]	3.2 [2.3–4.3]	5.2 [3.8–7.3]	< 0.001
sTfR, mg/L	2.7 (1.2)	3 (1.7)	2.5 (0.7)	2.6 (0.8)	< 0.001

Abbreviations: BMI, body mass index; eGFR, estimated glomerular filtration rate; EPO, erythropoietin; Hb, haemoglobin; HDL, high‐density lipoprotein; hs‐CRP, high sensitivity C‐reactive protein; MCV, mean corpuscular volume; SBP, systolic blood pressure; sTfR, soluble transferrin receptor; TSAT, transferrin saturation; UAE, urinary albumin excretion.

**TABLE 1B:** Baseline characteristics of the community‐dwelling individuals across tertiles of EPO levels.

		EPO tertiles (IU/L)			
		T1 < 6.5	T2: 6.5–9.2	T3 > 9.2	*p* for trend
Baseline characteristics					
Cancer incidence	18.5%	15.4%	18.6%	19.4%	< 0.001
Age, years	52.6 (11.9)	51.2 (11.4)	52.3 (11.7)	54.3 (12.3)	< 0.001
Male, %	49.2%	52.6%	50.2%	44.8%	< 0.001
Caucasian, %	96%	96.5%	96.1%	95.3%	0.063
High education, %	31.1%	33%	32.1%	28.4%	< 0.001
Current smoking, %	28.2%	32.3%	28.7%	23.5%	0.02
No alcoholic beverages, %	23.5%	19.6%	22.7%	28.2%	< 0.001
Clinical parameters					
BMI, kg/m^2^	26.7 (4.3)	26.1 (3.8)	26.4 (4.1)	27.5 (4.8)	< 0.001
SBP, mmHg	125.8 (18.6)	124.3 (17.8)	125.2 (18.7)	127.8 (19.3)	< 0.001
eGFR, mL/min/1.73 m^2^	94.3 [82–104.5]	96.2 [85–105.5]	94.7 [82.6–104.6]	91.8 [79.7–103.3]	< 0.001
UAE, mg/24 h	8.5 [6–14.8]	8.3 [6–13.7]	8.4 [6–14.4]	9 [6.1–17]	0.007
Hs‐CRP, mg/L	12.3 [0.6–2.9]	1.2 [0.6–2.6]	1.3 [0.6–2.8]	1.5 [0.7–3.5]	0.003
Serum protein, g/L	67.1 (4.1)	67.6 (4.1)	66.9 (4)	66.7 (4.1)	< 0.001
HDL, mg/dL	48.6 (12.1)	48.6 (11.9)	48.7 (11.9)	48.5 (12.3)	0.883
Total cholesterol, mmol/L	5.4 [4.7–6.1]	5.3 [4.8–6.2]	5.4 [4.7–6.1]	5.2 [4.6–6]	0.001
Comorbidities and medication use					
Hypertension, %	32.9%	27.9%	31.5%	39.2%	< 0.001
Use of antihypertensives, %	17.1%	13%	16.5%	21.9%	< 0.001
Use of lipid lowering drugs, %	9.4%	7.3%	8.8%	12%	< 0.001
Diabetes, %	5.9%	4%	5.2%	8.3%	< 0.001
Use of antidiabetic medication, %	1.2%	0.6%	1%	2.1%	< 0.001
Red blood cell and iron parameters					
Ferritin, μg/L	96 [47–171]	109 [56.5–182]	98.5 [51–174]	81 [32–153.8]	< 0.001
EPO, IU/L	7.76 [5.9–9.15]				
Hb, g/dL	14.1 (1.3)	14.4 (1.1)	14.2 (1.2)	13.8 (1.4)	< 0.001
MCV, fL	90.4 (4.6)	90.3 (3.9)	90.6 (4.1)	90.5 (5.7)	0.165
TSAT, %	25.1 (9.8)	26.5 (9.3)	25.6 (9.1)	23.2 (9.8)	< 0.001
sTfR, mg/L	2.7 (1.2)	2.4 (0.9)	2.5 (0.7)	3.1 (1.5)	< 0.001
Hepcidin, nM	3 [1.6–4.9]	3.4 [2–5.2]	3.1 [1.8–4.9]	2.6 [1.1–4.5]	< 0.001

Abbreviations: BMI, body mass index; eGFR, estimated glomerular filtration rate; EPO, erythropoietin; Hb, haemoglobin; HDL, high‐density lipoprotein; hs‐CRP, high sensitivity C‐reactive protein; MCV, mean corpuscular volume; SBP, systolic blood pressure; sTfR, soluble transferrin receptor; TSAT, transferrin saturation; UAE, urinary albumin excretion.

### 3.2 Iron Status, EPO Levels, and Risk of Overall Cancer

**FIGURE 1:**
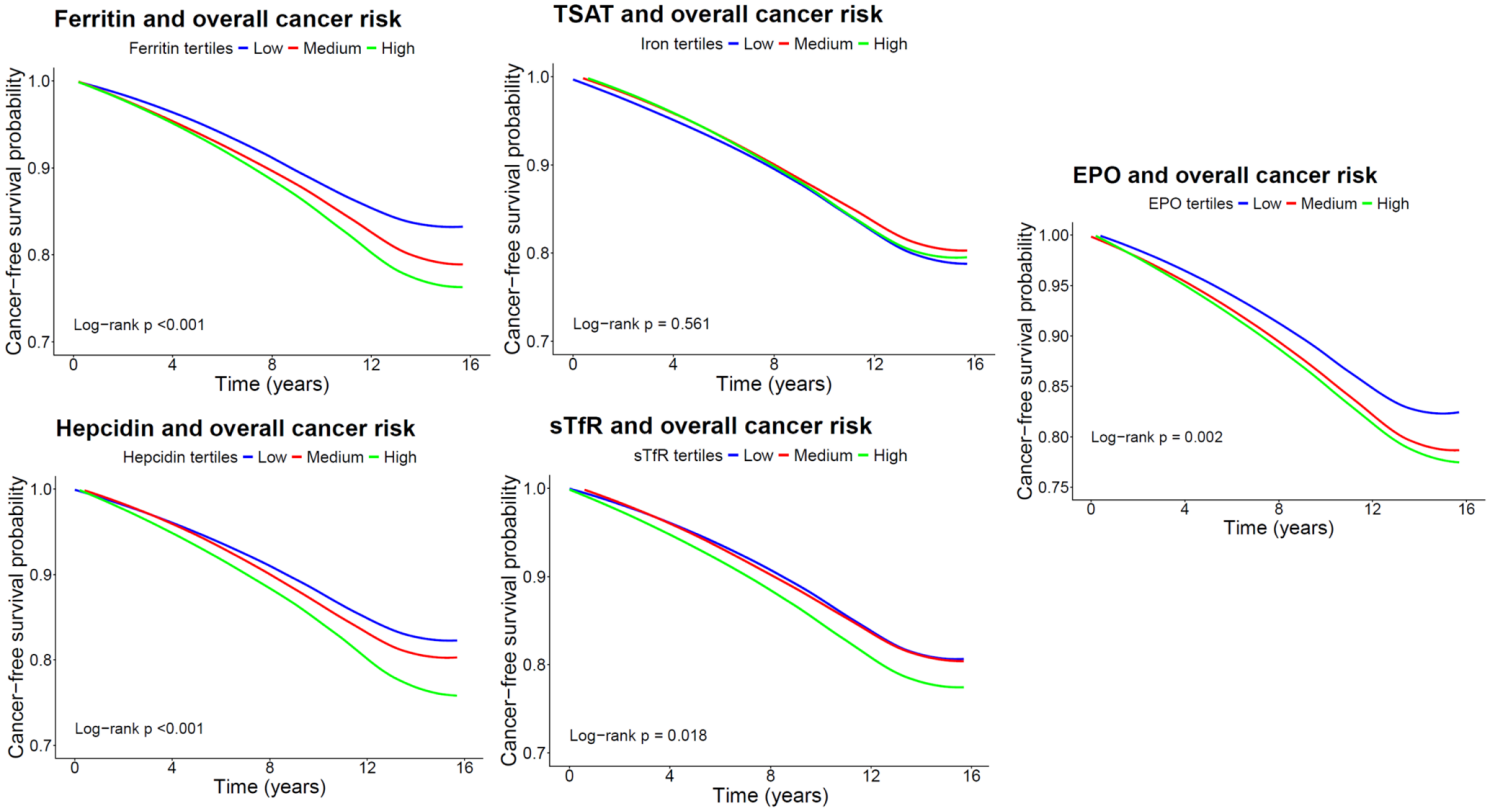
Kaplan–Meier curves depicting the univariable associations between iron status parameters and EPO levels on overall cancer risk. Survival is defined as the fraction of subjects that did not develop cancer during the total follow‐up. *p*‐values have been determined using the log‐rank test. EPO, erythropoietin; sTfR, soluble transferrin receptor; TSAT, transferrin saturation.

**FIGURE 2:**
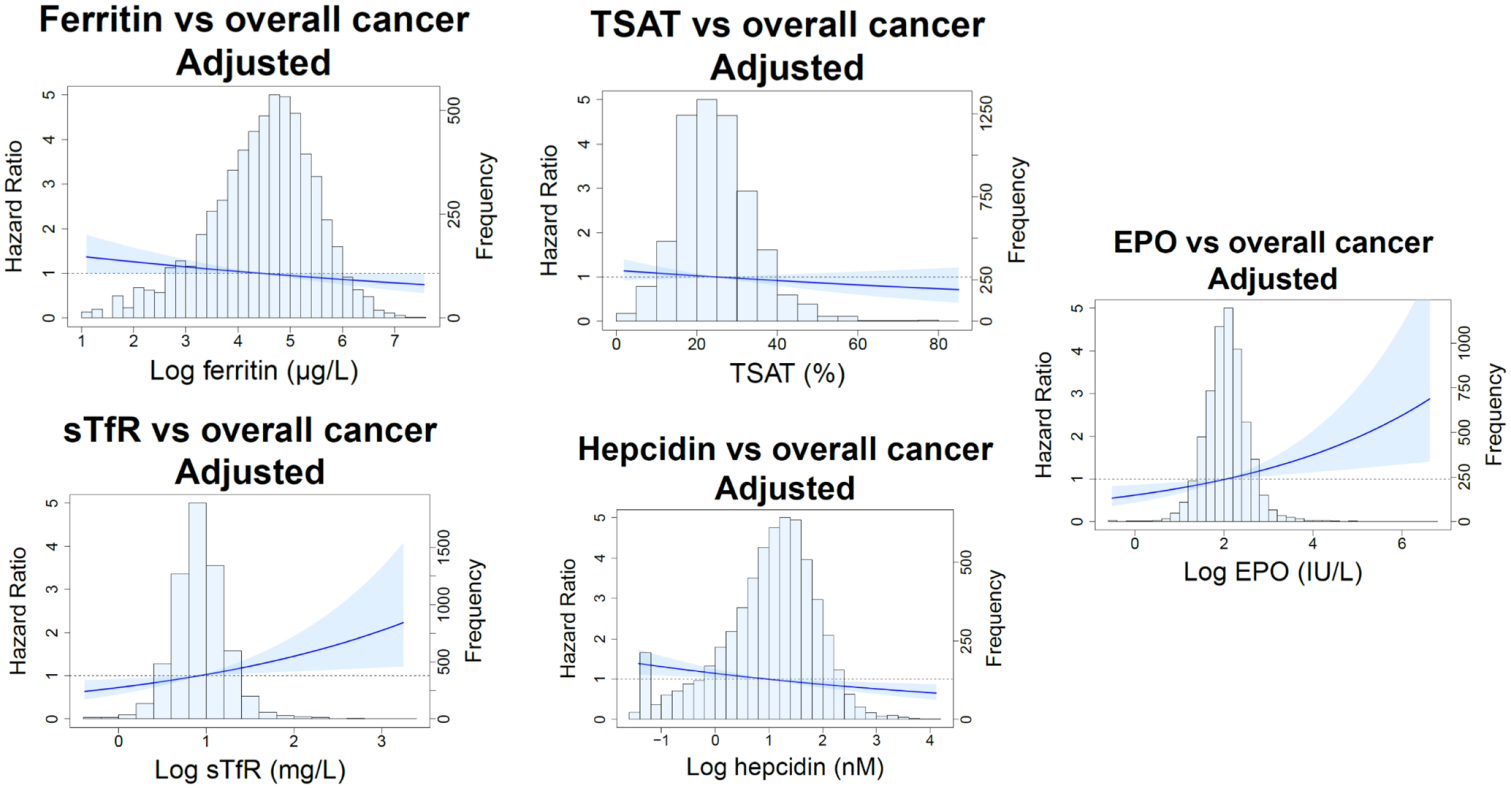
Restricted cubic splines depicting the hazard ratio between iron status parameters and erythropoietin and overall cancer risk (shaded areas indicate the 95% confidence interval). Hazard ratios are based on Cox proportional hazards regression models and adjusted for parameters in model 2. Ferritin, sTfR, hepcidin, and EPO were log‐transformed prior to analysis. The reference standard for log‐ferritin was 4.45 μg/L, 25% for TSAT, 0.9 mg/L for log‐sTfR, 3.79 nM for log‐hepcidin, and 2.06 lU/L for log‐EPO. EPO, erythropoietin; sTfR, soluble transferrin receptor; TSAT, transferrin saturation.

Over a median follow‐up time of 18.5 (IQR, 15.4–18.9) years, 1090 community‐dwelling individuals developed a de novo malignancy. Kaplan–Meier curves depicted in Figure 1 show the risk of developing overall cancer across tertiles of ferritin (*p* < 0.001), TSAT (*p* = 0.561), sTfR (*p* = 0.018), hepcidin (*p* < 0.001) and EPO levels (*p* = 0.002). In multivariable Cox proportional hazard regression analyses, each unit increase of log‐transformed sTfR (HR 1.35; 95% CI 1.01–1.80; *p =* 0.043) and log‐transformed EPO (HR 1.26; 95% CI 1.07–1.47; *p* = 0.005) was significantly associated with increased cancer risk, whereas each unit increase of log‐transformed hepcidin showed an inverse association with cancer risk (HR 0.88; 95% CI 0.80–0.96; *p* = 0.006). In contrast, no significant association with overall cancer was found per unit increase of log‐transformed ferritin and TSAT (Table 2; Tables S4–S8). The relationship between the different iron status parameters and EPO levels and overall cancer risk is visually depicted in Figure 2 by restricted cubic splines.

**TABLE 2:** Multivariate Cox proportional hazard analyses for the association of iron status parameters and EPO levels with overall cancer.

Parameter	HR (95% CI)	Tertiles of the different parameters		
		T1	T2	T3
Ferritin (μg/L)	0.92 (0.84–1.00)	1.00 (ref)	0.79 (0.64–0.96)*	0.80 (0.65–0.99)*
EPO (IU/L)	1.25 (1.07–1.47)**	1.00 (ref)	1.22 (1.00–1.50)*	1.22 (0.99–1.49)
TSAT (%)	0.99 (0.99–1.00)	1.00 (ref)	0.90 (0.74–1.10)	0.86 (0.71–1.05)
sTfR (mg/L)	1.39 (1.04–1.85)*	1.00 (ref)	0.95 (0.77–1.17)	1.14 (0.93–1.40)
Hepcidin (nM)	0.87 (0.79–0.95)***	1.00 (ref)	0.69 (0.56–0.84)***	0.77 (0.63–0.94)*

*Note:* Adjusted for race, sex, age, eGFR, BMI, smoking, alcohol, educational level and type 2 diabetes, diabetic medication, lipid lowering drugs, serum protein, hypertension, systolic blood pressure, UAE, hs‐CRP and total cholesterol.

Abbreviations: BMI, body mass index; eGFR, estimated glomerular filtration rate; EPO, erythropoietin; hs‐CRP, high sensitivity c‐reactive protein; sTfR, soluble transferrin receptor; TSAT, transferrin saturation; UAE, urinary albumin excretion.

*p < 0.05; **p < 0.01; ***p < 0.0625.

When analysing tertiles of the different biomarkers, we found that individuals in the middle and upper tertiles of both ferritin (HR: 0.78; 95% CI: 0.64–0.96; *p* = 0.02 and HR: 0.80; 95% CI: 0.65–0.99; *p* = 0.037, respectively) and hepcidin (HR: 0.69; 95% CI: 0.56–0.84; *p* < 0.001 and HR: 0.78; 95% CI: 0.64–0.95; *p* = 0.013) had a reduced risk of cancer compared to the lowest tertile in multivariable analyses. No significant associations were observed for tertiles of TSAT, sTfR, or EPO with overall cancer incidence (Table 2).

### 3.3 Iron Status, EPO Levels, and Site‐Specific Cancer Risk

We further explored the relationship between iron biomarkers and specific cancer types, focusing on those with an incidence above 0.5% in the cohort: skin, lung, kidney, breast, gastrointestinal (GI), prostate, and haematological cancers. In multivariable Cox proportional hazard regression analyses, higher hepcidin levels were associated with a decreased risk of GI cancer per log‐transformed unit increase (HR: 0.80; 95% CI: 0.68–0.99; *p* = 0.04, Table S7).

When examining site‐specific cancer risks across tertiles, decreased cancer risk associations were observed for higher ferritin levels; individuals in the middle tertile had a lower risk of developing urothelial cell carcinoma (HR: 0.49; 95% CI: 0.25–0.96; *p* = 0.043) and kidney cancer (HR: 0.37; 95% CI: 0.20–0.67; *p* = 0.002), as compared to the lowest tertile of ferritin (Table S4). Similarly, we observed that individuals in the highest tertile of TSAT had a significantly lower risk of haematological cancers compared to those in the lowest tertile (HR: 0.51; 95% CI: 0.28–0.95; *p* = 0.04, Table S5). Likewise, both the middle and highest tertiles of TSAT as well as the middle tertile of hepcidin were associated with reduced GI cancer risk (HR: 0.66; 95% CI: 0.45–0.97; *p* = 0.034, and HR: 0.61; 95% CI: 0.42–0.90; *p* = 0.014, and HR: 0.63; 95% CI: 0.42–0.95; *p =* 0.029 respectively, Tables S4–S8).

### 3.4 Sensitivity Analyses

We performed several sensitivity analyses. First, taking into account the possibility of reverse causation, we excluded individuals who had a cancer incidence within 1 year after baseline (Tables S9–S13). In this sensitivity analysis, the association between both log‐transformed hepcidin as a continuous variable (HR: 0.87; 95% CI: 0.79–0.95; *p* = 0.003) and the middle and highest tertile (HR: 0.68; 95% CI: 0.56–0.84; *p* ≤ 0.001 and HR: 0.77; 95% CI: 0.63–0.94; *p* = 0.013, respectively) remained significant. Additionally, the association between the middle and highest tertiles of ferritin and overall cancer remained significant (HR: 0.77; 95% CI: 0.62–0.95; *p* = 0.013 and HR: 0.76; 95% CI: 0.61–0.94; *p* = 0.011, respectively). Finally, the association between log‐transformed EPO as a continuous variable and overall cancer remained significant (HR: 1.23; 95% CI: 1.04–1.44; *p =* 0.016).

In site‐specific analysis, the association between the highest tertile of TSAT and the development of both GI and haematological cancers remained significant (HR: 0.65; 95% CI: 0.44–0.96; *p* = 0.033 and HR: 0.45; 95% CI: 0.23–0.87; *p* = 0.024, respectively). Additionally, individuals in the middle tertile of ferritin still had an increased risk for kidney cancer compared to those in the lowest tertile (HR: 0.4; 95% CI: 0.22–0.74; *p =* 0.005). Finally, the middle tertile of hepcidin remained significantly associated with the development of haematological cancers (HR: 0.46; 95% CI: 0.23–0.94; *p* = 0.041). Other associations between iron parameters and EPO and site‐specific cancer were not found to be significant following exclusion of cancer incidence within 1 year after baseline.

Second, we accounted for multiple testing in the site‐specific cancer analyses. When looking at site‐specific cancer risk, the relationship between kidney cancer and log‐transformed ferritin (as a continuous variable) remained significant (HR: 0.37, 95% CI: 0.2–0.67; *p* = 0.005), whereas the previously identified associations were not found to be statistically significant after adjusting for multiple testing.

**FIGURE 3:**
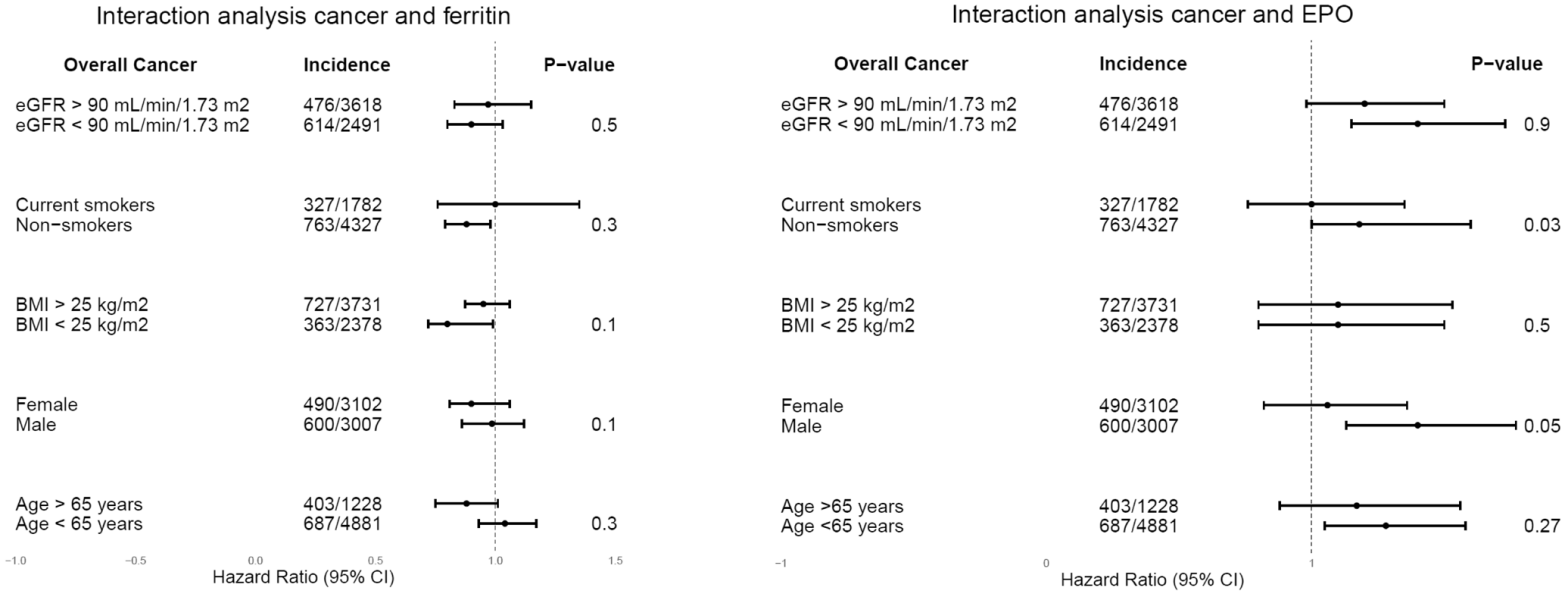
Forest plots showing effect modification between variables and ferritin and EPO levels on overall cancer risk. HRs and 95% CIs were derived from Cox proportional hazards regression models. HRs were adjusted for age, sex, BMI, smoking, alcohol, educational level, type 2 diabetes, and baseline eGFR. BMI, body mass index; CI, confidence interval; eGFR, estimated glomerular filtration rate; EPO, erythropoietin; HR, hazard ratio; sTfR, soluble transferrin receptor; TSAT, transferrin saturation; UAE, urinary albumin excretion.

We observed significant effect modification for hepcidin by sex (*p* = 0.048) and BMI (*p* = 0.03), and for sTfR and EPO by smoking status (*p* = 0.02 and *p* = 0.03, respectively; Figure 3, Figure S1, Table S14). When investigating the effect modification between hepcidin and sex, we found that in females, higher hepcidin levels were linked to reduced overall cancer risk (HR, 0.89; 95% CI: 0.82–0.98; *p* = 0.015), with the strongest association in the middle tertile (HR: 0.73; 95% CI: 0.55–0.98; *p* = 0.038; Table S14). In males, only the middle tertile of hepcidin showed a significantly lower cancer risk compared to the lowest tertile (HR, 0.73; 95% CI: 0.55–0.98; *p* = 0.045). We also found a significant effect modification between hepcidin and BMI. Specifically, in individuals with a BMI < 25 kg/m^2^, higher hepcidin levels were associated with reduced overall cancer risk (HR: 0.77; 95% CI: 0.66–0.90; *p* = 0.001), including breast cancer (HR, 0.63; 95% CI: 0.48–0.83; *p* = 0.002). Tertile analyses confirmed the latter association (middle: HR, 0.51; 95% CI: 0.27–0.97; *p* = 0.046; highest: HR: 0.35; 95% CI: 0.15–0.85; *p* = 0.025). No association was found in those with BMI > 25 kg/m^2^. Additionally, sTfR and EPO showed a significant effect modification with smoking. Among non‐smokers, elevated sTfR levels were linked to increased cancer risk (HR, 1.68; 95% CI: 1.21–2.32; *p* = 0.002), a pattern not observed in smokers (HR, 0.81; 95% CI: 0.47–1.39; *p* = 0.5). Similarly, higher EPO concentrations were associated with increased cancer risk in non‐smokers (HR: 1.40; 95% CI: 1.14–1.73; *p* = 0.002), including when EPO was categorized into tertiles (middle: HR, 1.5; 95% CI 1.15–1.97; *p* = 0.003 and highest: HR, 1.42; 95% CI 1.09–1.84; *p* = 0.007). Specifically, the middle EPO tertile was linked to increased kidney cancer risk (HR, 2.36; 95% CI: 1.12–4.97; *p* = 0.031; Table S14). No associations were found between EPO and cancer risk among smokers.

Finally, we assessed whether the commonly used cut‐offs of iron deficiency and iron overload in clinical practice were associated with an increased risk of overall cancer. Neither a ferritin < 30 μg/L nor a TSAT > 45% were associated with an increased risk for cancer development in adjusted analysis (HR: 1.27; 95% CI: 0.98–1.63; *p* = 0.07 and HR: 0.74; 95% CI 0.49–1.11; *p* = 0.14, respectively).

## 4 Discussion

In this study, we show that lower levels of hepcidin and elevated levels of EPO are associated with the development of cancer in community‐dwelling individuals. In addition, elevated levels of sTfR were suggestive of an association with the development of cancer over time. No association was found between cancer and other parameters of iron status. Excluding cancer diagnoses within 1 year of baseline attenuated the associations for sTfR; however, the inverse association of hepcidin and EPO on cancer risk remained evident in the tertile analyses. The current findings underscore the complex relationship between iron metabolism, EPO, and cancer, highlighting hepcidin and EPO as possible important factors in cancer development over time.

Cancer cells have a heightened need for iron [17]. Cancer cells often upregulate transferrin receptors on their surface to acquire iron more effectively [18]. Our study supports earlier studies which indicate elevated levels of sTfR as a factor associated with cancer development over time [10]. The tumour microenvironment is often hypoxic, which can further increase the demand for iron to support cell metabolism and survival [17]. Similar to findings by Zhang et al., higher sTfR levels may serve as an early warning signal for overall cancer risk [10]. We identified effect modifications between sTfR and smoking, where higher sTfR levels were associated with a strong significant risk for cancer in non‐smokers, whereas this association was not present in smokers. Smoking‐related inflammation and oxidative stress may obscure this relationship, whereas in non‐smokers, elevated sTfR may better reflect increased cellular iron demand linked to cancer risk. The fact that excluding the 1‐year follow‐up diagnoses of cancer incidence causes loss of the association between sTfR and overall cancer risk implies that it could be reverse causation, where increased sTfR levels might reflect early, undiagnosed cancer rather than being a true risk factor for developing cancer over time.

In addition, we found that increased EPO was associated with an increased risk for overall cancer. Although prospective studies for the association between EPO and cancer development are scarce, the use of erythropoietin stimulating agents has been associated with an increased risk for cancer development [19]. This may be related to enhanced angiogenesis, lymphangiogenesis and cellular proliferation, as well as the expression of EPO receptors on various tumours [19, 20]. We observed effect modification by smoking where higher EPO concentrations were associated with increased cancer risk in non‐smokers but not in smokers. In non‐smokers, elevated EPO may reflect mild hypoxia or inflammation that supports tumour growth and survival, whereas in smokers, the strong carcinogenic effects of smoking may overshadow the influence of EPO and mask this association. Additionally, smoking may disrupt EPO regulation, making its levels less indicative of cancer risk in this group. The observed associations between sTfR, EPO, and smoking warrant caution when interpreting these iron‐related biomarkers in smokers in a clinical context. Elevated sTfR and EPO levels in smokers may reflect smoking‐induced tissue hypoxia and compensatory erythropoietic stimulation, rather than underlying malignancy.

We found high hepcidin levels to be inversely related to cancer development. Hepcidin causes the degradation of ferroportin [21]. Therefore, increased hepcidin might result in a decreased available iron concentration. This decreased iron availability could result in decreased iron‐related tumorigenic cellular damage by less reactive oxygen species formation, modulation of tumour microenvironment and angiogenesis, and directly blocking the iron availability for cancer cells. This all would result in a decreased tumour risk. Furthermore, we observed effect modification with hepcidin and sex and BMI. Most notably, in individuals with a BMI < 25 kg/m^2^, higher hepcidin was strongly associated with a decreased risk for cancer, whereas this was not observed in those with a BMI > 25 kg/m^2^. Possibly, obesity‐related inflammation could elevate hepcidin independently of iron homeostasis [22]. Although we adjusted for hs‐CRP levels, obesity is characterized by a complex inflammatory profile, and several pro‐inflammatory cytokines that are not fully captured by hs‐CRP measurements may remain elevated and contribute to the observed associations [23, 24]. The reason that our result of hepcidin is in contrast with some previous studies which found high hepcidin levels to be associated with colorectal cancer and pancreatic cancer could in fact be due to the inflammatory environment that accompanies tumour growth [25]. The use of hepcidin antagonists has been implicated in a range of conditions, such as anaemia associated with inflammation, chronic kidney disease and cancers, without phase 3 clinical trial evidence yet [26]. More studies are needed to study the applicability of these drugs in clinical practice.

Of note, low iron status can also be a reflection of occult blood loss, which might imply the presence of early stage tumours. In the current study, we did not identify an overall association between ferritin and TSAT with overall cancer development. The suggestive association in the current study of lower GI cancer risk with higher TSAT levels and urothelial cell carcinoma and renal cell carcinoma with ferritin is most likely the reflection of (occult) blood loss through the digestive system, which is also reflected by the fact that sensitivity analysis showed no significant association between TSAT and GI cancer, or ferritin and urothelial cell carcinoma when removing cancer diagnosis within the first year after blood sampling. Although the association between ferritin and renal cell carcinoma was still significant, it was highly attenuated following removal of tumours diagnosed within the first year after blood sampling.

Our study has several strengths and limitations. To our knowledge, this is the first prospective study investigating such an extensive set of iron parameters, including sTfR, hepcidin, and EPO with cancer incidence. A major strength is our well‐phenotyped cohort, which allowed adjustment for important lifestyle and socioeconomic covariates and that extensive data was available on cancer due to the long follow‐up of the PREVEND study. Another strength is the unbiased ascertainment of cancer incidence through linkage with the Palga registry, which has complete national coverage. A first limitation could be that the PREVEND study contains primarily individuals with a low urinary albumin excretion at baseline, which could have resulted in inclusion bias. Secondly, the absence of a second measurement moment for iron and haematological parameters means limited correction for the fluctuation of iron homeostasis over time. However, it should be realized that most epidemiologic studies use a single baseline measurement for studying the association of variables with outcomes, which adversely affects the strength and significance of the association of these variables with outcomes. Thirdly, the cohort primarily consisted of Caucasians in the North of the Netherlands, which may limit generalizability [27].

In conclusion, hepcidin levels were consistently found to be potentially protective against cancer development by diminishing iron availability for cancer cells, whereas elevated EPO concentrations showed increased risk for cancer development. Additionally, elevated sTfR was suggestive of an association with increased risk of cancer in community‐dwelling individuals. The fact that the latter results were annihilated after excluding individuals with 1‐year follow‐up suggests possible reverse causation, potentially rendering sTfR as a marker for occult cancer. These findings highlight the complex interplay between iron metabolism, EPO, and cancer, and future studies should further investigate underlying mechanisms and unravel whether these parameters can be used in predictive models or as screening tools to identify cancers at early stages.

## Author Contributions

**Siem J. van Alfen:** conceptualization; formal analysis, methodology, software, visualization, writing – original draft preparation. **Pien Rawee:** software; writing – review and editing. **Ilja M. Nolte:** software; writing – review and editing. **Li Luo:** writing – review and editing. **Bert van der Vegt:** data curation; investigation; writing – review and editing. **Jenny E. Kootstra‐Ros:** data curation; investigation; writing – review and editing. **Stephan J. L. Bakker:** writing – review and editing. **Ron T. Gansevoort:** writing – review and editing. **Thera P. Links:** writing – review and editing. **Wouter T. Zandee:** writing – review and editing. **Michele F. Eisenga:** conceptualization; formal analysis; methodology; software; supervision; visualization; writing – original draft preparation.

## Funding

The PREVEND study is supported by several grants from the Dutch Kidney Foundation (Nierstichting) (E.033) and the Dutch Heart Foundation (Hartstichting) (2001.005), the Dutch Government Ministry of Health, Welfare and Sport (Ministerie van Volksgezondheid, Welzijn en Sport), the National Institutes of Health, and the Universitair Medisch Centrum Groningen, the Netherlands.

## Ethics Statement

The PREVEND study conformed to the principles drafted in the Helsinki declaration. Local medical ethics committee approval was obtained (approval number: MEC96/01/022), and informed consent was provided by all participants.

## Conflicts of Interest

Dr. Eisenga has received speaker/consultancy fees from Vifor Pharma, Astellas, Cablon Medical, GlaxoSmithKline, Medice and Pharmacosmos; has served on the Advisory Board for Cablon Medical, GlaxoSmithKline and Medice; and has received grant support from Cablon Medical and Astellas (all to employer in all instances). Dr. Van der Vegt reports honoraria received by UMCG for expertise or scientific advisory board/consultancy (on request): Visiopharm, Philips, MSD/Merck, Daiichi‐Sankyo/AstraZenica; Speaker's fee MSD/Merck. Unrestricted research grants from OWKIN, GE Healthcare, Roche, IBEX. Personal fees from DEKRA. All unrelated to the current manuscript. The other authors report no conflicts of interest.

## Supporting information


**Table S1:** Baseline characteristics of the community‐dwelling individuals across quartiles of TSAT levels.
**Table S2:** Baseline characteristics of the community‐dwelling individuals across quartiles of sTfR levels.
**Table S3:** Baseline characteristics of the community‐dwelling individuals across quartiles of hepcidin levels.
**Table S4:** Association of ferritin with cancer development.
**Table S5:** Association of TSAT with cancer development.
**Table S6:** Association of sTfR with cancer development.
**Table S7:** Association of hepcidin with cancer development.
**Table S8:** Association of EPO with cancer development.
**Table S9:** Association of ferritin with cancer development for cancers diagnosed 1 year post blood sampling.
**Table S10:** Association of TSAT with cancer development for cancers diagnosed 1 year post blood sampling.
**Table S11:** Association of sTfR with cancer development for cancers diagnosed 1 year post blood sampling.
**Table S12:** Association of hepcidin with cancer development for cancers diagnosed 1 year post blood sampling.
**Table S13:** Association of EPO with cancer development for cancers diagnosed 1 year post blood sampling.
**Table S14:** Association of iron parameters and EPO levels with significant effect modifications with cancer development over time.
**Figure S1:** Forest plot showing effect modification by different subgroups on the cancer incidence for TSAT, sTfR and hepcidin.

## Data Availability

The data underlying this article will be shared on reasonable request to the corresponding author.
