# Supplementary material for: Iron Status, Erythropoietin, and Cancer Incidence in the General Population
Source: Eur J Clin Invest. 2026 May 8;56:e70218. doi: 10.1111/eci.70218 (PMC13154720; doi:10.1111/eci.70218)
Supplement: Supplementary file 1 — Table S1: Baseline characteristics of the community‐dwelling individuals across quartiles of TSAT levels. Table S2: Baseline characteristics of the community‐dwelling individuals across quartiles of sTfR levels. Table S3: Baseline characteristics of the community‐dwelling individuals across quartiles of hepcidin levels. Table S4: Association of ferritin with cancer development. Table S5: Association of TSAT with cancer development. Table S6: Association of sTfR with cancer development. Table S7: Association of hepcidin with cancer development. Table S8: Association of EPO with cancer development. Table S9: Association of ferritin with cancer development for cancers diagnosed 1 year post blood sampling. Table S10: Association of TSAT with cancer development for cancers diagnosed 1 year post blood sampling. Table S11: Association of sTfR with cancer development for cancers diagnosed 1 year post blood sampling. Table S12: Association of hepcidin with cancer development for cancers diagnosed 1 year post blood sampling. Table S13: Association of EPO with cancer development for cancers diagnosed 1 year post blood sampling. Table S14: Association of iron parameters and EPO levels with significant effect modifications with cancer development over time. Figure S1: Forest plot showing effect modification by different subgroups on the cancer incidence for TSAT, sTfR and hepcidin. [file ECI-56-e70218-s001.docx]

*Online Supplementary Material*

**Iron Status, Erythropoietin, and Cancer Incidence in the General Population**Siem J. van Alfen, BsC^1^, Pien Rawee, MSc^2^, Li Luo, MD^2^, Ilja M. Nolte, PhD^3^, Bert van der Vegt, MD,PhD^4^, Jenny E. Kootstra-Ros, PharmD, PhD^5^, Stephan J.L. Bakker, MD, PhD^2^, Ron T. Gansevoort, MD, PhD^2^, Thera P. Links, MD, PhD^1^, Wouter T. Zandee, MD, PhD^1^, Michele F. Eisenga, MD, PhD^2^

^1^Division of Endocrinology, Department of Internal Medicine, University Medical Center Groningen, University of Groningen, Groningen, the Netherlands

^2^Division of Nephrology, Department of Internal Medicine, University Medical Center Groningen, University of Groningen, Groningen, the Netherlands

^3^Department of Epidemiology, University Medical Center Groningen, University of Groningen, Groningen, the Netherlands
^4^Department of Pathology, University Medical Center Groningen, University of Groningen, Groningen, the Netherlands

^5^Department of Laboratory Medicine, University Medical Center Groningen, University of Groningen, Groningen, the Netherlands

**Table of contents:**

**Methods M1:** Description of analytical methods used to measure iron-related biomarkers, EPO and other covariates.

**Table S1-3** baseline tables of TSAT, sTfR and hepcidin

**Table S4-8** Univariable and adjusted Cox proportional hazards analysis for the association between iron parameters with overall cancer and site-specific cancer outcomes.

**Table S9-13** Univariate and adjusted Cox proportional hazards analysis for the association between iron parameters with overall cancer and site-specific cancer outcomes with the exclusion of patients who developed cancer within the first year post blood sampling

**Table S14** Association of iron parameters with significant effect modifications with cancer development.

**Figure S1** Forest plot showing effect modification by different subgroups on the cancer incidence

**Methods M1: Description of analytical methods used to measure iron-related biomarkers and other covariates.**

Fasting blood samples were drawn in the morning from all subjects from April 24, 2001, to December 3, 2003. All hematologic parameters were measured in fresh venous blood. Serum creatinine was measured using an enzymatic method on a Roche Modular analyzer (Roche Diagnostics, Mannheim, Germany). For predicting the estimated glomerular filtration rate (eGFR), the Chronic Kidney Disease Epidemiology Collaboration was applied.^1^ UAC was measured by nephelometry with a threshold of 2.3 mg/L, and intra-assay and inter-assay coefficients of variation of 2.2% and 2.6%, respectively (Dade Behring Diagnostic, Marburg, Germany). Serum iron was measured using a colorimetric assay, ferritin using immunoassay, and transferrin using an immunoturbidimetric assay, Soluble transferrin receptor (sTfR) was measured using an automated homogenous immunoturbidimetric assay with intra- and interassay CVs <2% and <5% (all Roche Diagnostics).^2^ Transferrin saturation (TSAT, %) was calculated as 100 × serum iron (μmol/L) ÷ 25 × transferrin (g/L).^3^ Serum EPO was measured using an immunoassay based on chemiluminescence (Immulite EPO assay, DPC, Los Angeles, CA). Serum hepcidin was measured with a competitive enzyme-linked immunosorbent assay, as described elsewhere with intra- and interassay coefficients of variation of 8.6% and 16.2%, respectively.^4^ High-sensitivity C-reactive protein (hs-CRP), total cholesterol, and high density lipids, and total protein levels were measured using routine laboratory procedures.

**References**

1. Levey AS, Stevens LA, Schmid CH, et al. A New Equation to Estimate Glomerular Filtration Rate. *Ann Intern Med*. 2009;150(9):604-612. doi:10.7326/0003-4819-150-9-200905050-00006

2. Pfeiffer CM, Cook JD, Mei Z, Cogswell ME, Looker AC, Lacher DA. Evaluation of an automated soluble transferrin receptor (sTfR) assay on the Roche Hitachi analyzer and its comparison to two ELISA assays. *Clinica Chimica Acta*. 2007;382(1-2):112-116. doi:10.1016/j.cca.2007.04.008

3. Mercadal L, Metzger M, Haymann JP, et al. A 3-marker index improves the identification of iron disorders in CKD anaemia. *PLoS One*. 2014;9(2). doi:10.1371/journal.pone.0084144

4. Kroot JJC, Laarakkers CMM, Geurts-Moespot AJ, et al. Immunochemical and mass-spectrometry-based serum hepcidin assays for iron metabolism disorders. *Clin Chem*. 2010;56(10):1570-1579. doi:10.1373/clinchem.2010.149187

.

**Table S1| Baseline characteristics of the community-dwelling individuals across quartiles of TSAT levels**

|  |  | TSAT tertiles (%) | | |  | |
| --- | --- | --- | --- | --- | --- | --- |
|  |  | **T1: < 20.1** | **T2: 20.1-27.8** | **T3: >27.8** | | ***P for trend*** |
| Baseline characteristics | | | | | | |
| Cancer incidence | 18.8% | 18.6% | 16.9% | 18% | | 0.6 |
| Age, y | 52.7 (11.9) | 51.9 (11.7) | 53.3 (11.9) | 52.8 (12.1) | | 0.017 |
| Male, % | 49.2% | 38.2% | 50.2% | 59.2% | | <0.001 |
| Caucasian, % | 96% | 95.3% | 95.9% | 96.6% | | 0.037 |
| High education, % | 31% | 29.7% | 31.2% | 32.2% | | 0.044 |
| Current smoking, % | 28.3% | 27.1% | 26.4% | 31.2% | | 0.323 |
| No alcoholic beverages, % | 23.7% | 29.7% | 31.2% | 32.2% | | <0.001 |
| Clinical parameters | | | | | | |
| BMI, kg/m^2 | 26.7 (4.4) | 27.2 (4.7) | 26.7 (4.3) | 26.2 (3.9) | | <0.001 |
| SBP, mmHg | 125.9 (18.7) | 125.5 (18.8) | 126.3 (18.6) | 125.9 (18.7) | | 0.6 |
| eGFR, mL/min/1.73 m^2 | 94.2 [81.9-104.5] | 94.6 [81.9-104.6] | 93.5 [81.2-104.5] | 94.8 [82.6-104.3] | | 0.611 |
| UAE, mg/24h | 8.5 [6-15.1 | 8.6 [6-15.3] | 8.4 [6-15] | 8.6 [6-15] | | 0.523 |
| Hs-CRP, mg/L | 1.3 [0.6-3] | 1.7 [0.7-4] | 1.3 [0.6-2.9] | 1.1 [0.5-2.4] | | <0.001 |
| Serum protein, g/dL | 67.1 (4.1) | 67.3 (4.2) | 67 (4) | 66.9 (4.1) | | 0.002 |
| HDL, mg/dL | 48.5 (12.1) | 48.8 (12.4) | 48.4 (12.1) | 48.4 (11.9) | | 0.301 |
| Total cholesterol, mmol/L | 5.4 [4.7-6.1] | 5.3 [4.7-6.1] | 5.4 [4.8-6.2] | 5.3 [4.7-6.1 | | 0.452 |
| Comorbidities and medication use | | | | | | |
| Hypertension, % | 33.3% | 35.4% | 34.4% | 30.2% | | <0.001 |
| Use of antihypertensives, % | 17.5% | 20.1% | 18% | 14.3% | | <0.001 |
| Use of lipid lowering drugs, % | 9.4% | 9.6% | 10% | 8.7% | | 0.327 |
| Diabetes, % | 5.9% | 7% | 5.9% | 5% | | 0.008 |
| Use of antidiabetic medication, % | 1.2% | 1.9% | 0.9% | 0.8% | | 0.003 |
| Iron parameters | | | | | | |
| Ferritin, µg/L | 96 [47-171] | 63 [26-124] | 105 [55.5-178] | 127 [66-217 | | <0.001 |
| EPO, U/L | 7.76 [5.9-9.15] | 8.5 [6.3-11.6] | 7.6 [5.9-9.8] | 7.4 [5.6-9.6] | | <0.001 |
| Hb, g/dL | 14.1 (1.3) | 13.7 (1.4) | 14.3 (1.1) | 14.5 (1.1) | | <0.001 |
| MCV, fl | 90.4 (4.6) | 89 (5.3) | 90.6 (3.9) | 91.7 (4.2) | | <0.001 |
| TSAT, % | 25.1 (9.8) | 15.5 (4.1) | 24.3 (2.1) | 35.4 (7.2) | | <0.001 |
| Hepcidin, nM | 3 [1.6-4.9] | 2.3 [1-1.39] | 3.2 [1.8-4.9] | 3.6 [2.2-5.7 | | <0.001 |
| sTfR, mg/L | 2.7 (1.2) | 3.1 (1.7) | 2.6 (0.7) | 2.4 (0.7) | | <0.001 |

*P* for trend is determined by a χ^2^ test (categorical variables), or by linear regression or a Kruskal–Wallis test (continuous variables). Normally distributed data are presented as mean ± standard deviation, skewed data as median [ interquartile range] and categorical data as nunmber (valid percentage).

Abbreviations: BMI, body mass index; CRP, C-reactive protein; SBP, systolic blood pressure; eGFR, estimated glomerular filtration rate; UAE, urinary albumin excretion; HDL, high-density lipoprotein; hs-CRP, high sensitivity C-reactive protein; , hemoglobin; MCV, mean corpsular volume; TSAT, transferrin saturation; sTfR, soluble transferrin receptor; EPO, erythropoietin.

|  |  | sTfR tertiles (mg/L) | | | |  |
| --- | --- | --- | --- | --- | --- | --- |
|  |  | **T1: < 2.2** | **T2: 2.2-2.8** | **T3: > 2.8** | ***P for trend*** |  |
| Baseline characteristics | | | | | | |
| Cancer incidence | 15.4% | 16.7% | 17.3% | 19.5% | 0.033 |  |
| Age, y | 52.4 (11.8) | 50.9 (10.7) | 52.7 (12.1) | 53.6 (12.5) | <0.001 |  |
| Male, % | 49.9% | 47.8% | 50.2% | 51.5% | 0.34 |  |
| Caucasian, % | 95.8% | 97.3% | 96.3% | 93.8% | <0.001 |  |
| High education, % | 31.3% | 34.1% | 31.1% | 28.8% | <0.001 |  |
| Current smoking, % | 28.2% | 38.7% | 26.8% | 19.3% | <0.001 |  |
| No alcoholic beverages, % | 23.8% | 18.7% | 22.9% | 29.8% | <0.001 |  |
| Clinical parameters | | | | | | |
| BMI, kg/m^2 | 26.7 (4.4) | 26 (4) | 26.8 (4.3) | 27.4 (4.6 | <0.001 |  |
| SBP, mmHg | 125.8 (18.5) | 122.7 (17.2) | 126.2 (18.4) | 128.4 (19.5) | <0.001 |  |
| eGFR, mL/min/1.73 m^2 | 94.7 [82.2-105.6] | 97.2 [86.6 – 105.9] | 94.4 [81.6 – 104.5] | 91.5 [79.4-103.1] | <0.001 |  |
| UAE, mg/24h | 8.5 [6-15] | 8.2 [5.9-13.7] | 8.5 [6.1-14.6] | 8.9 [6.1-17.3] | <0.001 |  |
| Hs-CRP, mg/L | 1.3 [0.6-3] | 1.1 [0.5-2.7] | 1.3 [0.6-2.8] | 1.5 [0.7-3.4] | 0.003 |  |
| Serum protein, g/L | 67.1 (4.1) | 66.1 (4) | 67.2 (3.9) | 67.9 (4.2) | <0.001 |  |
| HDL, mg/dL | 48.5 (12.2) | 49.3 (11.9) | 48.4 (12.2) | 47.9 (12.6) | 0.023 |  |
| Total cholesterol, mmol/L | 5.3 [4.7-6.1] | 5.3 [4.6-6] | 5.3 [4.7-6.1] | 5.4 [4.7-6.2 | 0.001 |  |
| Comorbidities and medication use | | | | | | |
| Hypertension, % | 33% | 27.5% | 32.4% | 39% | <0.001 |  |
| Use of antihypertensives, % | 17% | 14% | 16.4% | 20.5% | <0.001 |  |
| Use of lipid lowering drugs, % | 9.1% | 7.8% | 8.9% | 10.6% | 0.005 |  |
| Diabetes, % | 5.7% | 5.2% | 5.5% | 6.5% | 0.098 |  |
| Use of antidiabetic medication, % | 1.2% | 1.1% | 0.9% | 1.6% | 0.197 |  |
| Iron parameters | | | | | | |
| Ferritin, µg/L | 96 [47-171] | 101 [55-174.8] | 103 [52-174.8 | 81 [30-164] | <0.001 |  |
| EPO, IU/L | 7.7 [5.9-10.3] | 7 [5.4-8.9] | 7.5 [5.8-9.7] | 9 [6.8-12.4] | <0.001 |  |
| Hb, g/dL | 14.2 (1.3) | 14.1 (1.1) | 14.3 (1.1) | 14.1 (1.5) | 0.278 |  |
| MCV, fl | 90.4 (4.7) | 91.4 (3.8) | 90.7 (4) | 89 (5.6) | <0.001 |  |
| TSAT, % | 25.1 (9.5) | 28.1 (9.5) | 25.7 (8.8) | 21.3 (9) | <0.001 |  |
| Hepcidin, nM | 3 [1.6-4.9] | 3.3 [1.9-5.1] | 3.2 [1.9-5.1] | 2.7 [1.2-4.6] | <0.001 |  |
| sTfR, mg/L | 2.7 (1.2) | 1.9 (0.3) | 2.5 (0.2) | 3.7 (1.6) | <0.001 |  |

**Table S2|** Baseline characteristics of the community-dwelling individuals across quartiles of sTfR levels

*P* for trend is determined by a χ^2^ test (categorical variables), or by linear regression or a Kruskal–Wallis test (continuous variables). Normally distributed data are presented as mean ± standard deviation, skewed data as median [ interquartile range] and categorical data as nunmber (valid percentage).

Abbreviations: BMI, body mass index; CRP, C-reactive protein; SBP, systolic blood pressure; eGFR, estimated glomerular filtration rate; UAE, urinary albumin excretion; HDL, high-density lipoprotein; hs-CRP, high sensitivity C-reactive protein; , hemoglobin; MCV, mean corpsular volume; TSAT, transferrin saturation; sTfR, soluble transferrin receptor; EPO, erythropoietin.

**Table S3|** Baseline characteristics of the community-dwelling individuals across quartiles of hepcidin levels

|  |  | Hepcidin tertiles (nM) | | | |
| --- | --- | --- | --- | --- | --- |
|  |  | **T1: <2.1** | **T2: 2.1-4.1** | **T3: > 4.1** | ***P for trend*** |
| Baseline characteristics | | | | | |
| Cancer incidence | 18.6% | 15.6% | 17.5% | 20.7% | <0.001 |
| Age, y | 52.6 (11.9) | 51.2 (11.4) | 52.3 (11.7) | 54.3 (12.3) | <0.001 |
| Male, % | 49.2% | 52.6% | 50.2% | 44.8% | <0.001 |
| Caucasian, % | 96% | 96.5% | 96.1% | 95.3% | 0.168 |
| High education, % | 31.1% | 33% | 32.1% | 28.4% | <0.001 |
| Current smoking, % | 28.2% | 32.3% | 28.7% | 23.5% | <0.001 |
| No alcoholic beverages, % | 23.5% | 19.6% | 22.7% | 28.2% | 0.011 |
| Clinical parameters | | | | | |
| BMI, kg/m^2 | 26.7 (4.3) | 26.1 (3.8) | 26.4 (4.1) | 27.5 (4.8) | <0.001 |
| SBP, mmHg | 125.8 (18.6) | 124.3 (17.8) | 125.2 (18.7) | 127.8 (19.3) | <0.001 |
| eGFR, mL/min/1.73 m^2 | 94.3 [82-104.5] | 96.2 [85-105.5] | 94.7 [82.6-104.6] | 91.8 [79.7-103.3] | <0.001 |
| UAE, mg/24h | 8.5 [6-14.8] | 8.3 [6-13.7] | 8.4 [6-14.4] | 9 [6.1-17] | 0.004 |
| Hs-CRP, mg/L | 12.3 [0.6-2.9] | 1.2 [0.6-2.6] | 1.3 [0.6-2.8] | 1.5 [0.7-3.5] | <0.001 |
| Serum protein, g/L | 67.1 (4.1) | 66.3 (4.1) | 67 (4) | 67.8 (4.1) | <0.001 |
| HDL, mg/dL | 48.6 (12.1) | 48.6 (11.9) | 48.7 (11.9) | 48.5 (12.3) | <0.001 |
| Total cholesterol, mmol/L | 5.4 [4.7-6.1] | 5.3 [4.8-6.2] | 5.4 [4.7-6.1] | 5.2 [4.6-6] | <0.001 |
| Comorbidities and medication use | | | | | |
| Hypertension | 33.4% | 24% | 33.6% | 42.6% | <0.001 |
| Use of antihypertensives, % | 17.1% | 13% | 16.5% | 21.9% | <0.001 |
| Use of lipid lowering drugs, % | 9.4% | 7.3% | 8.8% | 12% | <0.001 |
| Diabetes, % | 5.9% | 4% | 5.2% | 8.3% | <0.001 |
| Use of antidiabetic medication, % | 1.2% | 0.6% | 1% | 2.1% | 0.101 |
| Iron parameters | | | | | |
| Ferritin, µg/L | 96 [47-171] | 109 [56.5-182] | 98.5 [51-174] | 81 [32-153.8] | <0.001 |
| EPO, IU/L | 7.8 [5.9-10.3] | 8.6 [6.4-11.8] | 7.6 [5.8-10] | 7.3 [5.6-9.5] | <0.001 |
| Hb, g/dL | 14.1 (1.3) | 14.4 (1.1) | 14.2 (1.2) | 13.8 (1.4) | <0.001 |
| MCV, fl | 90.4 (4.6) | 90.3 (3.9) | 90.6 (4.1) | 90.5 (5.7) | <0.001 |
| TSAT, % | 25.1 (9.8) | 26.5 (9.3) | 25.6 (9.1) | 23.2 (9.8) | <0.001 |
| Hepcidin, nM | 3 [1.6-4.9] | 1.16 [0.61–1.64] | 3.01 [2.56–3.55] | 5.96 [4.87–7.95] | <0.001 |
| sTfR, mg/L | 2.7 (1.2) | 3 (1.7) | 2.5 (0.7) | 2.5 (0.8) | <0.001 |

*P* for trend is determined by a χ^2^ test (categorical variables), or by linear regression or a Kruskal–Wallis test (continuous variables). Normally distributed data are presented as mean ± standard deviation, skewed data as median [ interquartile range] and categorical data as nunmber (valid percentage).

Abbreviations: BMI, body mass index; CRP, C-reactive protein; SBP, systolic blood pressure; eGFR, estimated glomerular filtration rate; UAE, urinary albumin excretion; HDL, high-density lipoprotein; hs-CRP, high sensitivity C-reactive protein; , hemoglobin; MCV, mean corpsular volume; TSAT, transferrin saturation; sTfR, soluble transferrin receptor; EPO, erythropoietin.

**Table S4|** Association of ferritin with cancer development

|  |  | Tertiles of ferritin | | |
| --- | --- | --- | --- | --- |
|  | **Ferritin as continuous variable** | **T1: < 61 µg/L** | **T2: 61-139 µg/L** | **T3: >139 µg/L** |
| Overall cancer |  |  |  |  |
| No. of events/subjects | 645/6109 | 191/2043 | 207/2030 | 247/2036 |
| Univariate | 1.13 (1.05–1.22) *** | 1.00 (ref) | 1.10 (0.90–1.34) | 1.32 (1.09–1.6) *** |
| Model 1 | 0.92 (0.84–1.00) | 1.00 (ref) | 0.80 (0.65–0.98) * | 0.83 (0.68–1.02) |
| Model 2 | 0.92 (0.84–1.00) | 1.00 (ref) | 0.78 (0.64–0.96) * | 0.80 (0.65–0.99) * |
| Skin cancer |  |  |  |  |
| No. of events/subjects | 445/6109 | 111/2043 | 165/2030 | 169/2036 |
| Univariate | 1.21 (1.10–1.33) *** | 1.00 (ref) | 1.53 (1.20–1.95) *** | 1.59 (1.25–2.02) *** |
| Model 1 | 0.98 (0.88–1.09) | 1.00 (ref) | 1.08 (0.85–1.38) | 0.99 (0.77–1.28) |
| Model 2 | 1.01 (0.90–1.13) | 1.00 (ref) | 1.11 (0.86–1.43) | 1.05 (0.81–1.38) |
| Lung cancer |  |  |  |  |
| No. of events/subjects | 108/6109 | 26/2043 | 31/2030 | 51/2036 |
| Univariate | 1.57 (1.28–1.92) *** | 1.00 (ref) | 1.21 (0.72–2.04) | 2.00 (1.25–3.21) *** |
| Model 1 | 1.17 (0.93–1.46) | 1.00 (ref) | 0.75 (0.44–1.26) | 0.97 (0.59–1.58) |
| Model 2 | 1.06 (0.84–1.33) | 1.00 (ref) | 0.66 (0.39–1.13) | 0.77 (0.46–1.29) |
| Kidney cancer |  |  |  |  |
| No. of events/subjects | 99/6109 | 28/2043 | 19/2030 | 52/2036 |
| Univariate | 1.40 (1.14–1.73) *** | 1.00 (ref) | 0.69 (0.39–1.24) | 1.92 (1.21–3.03) ** |
| Model 1 | 0.96 (0.77–1.21) | 1.00 (ref) | 0.39 (0.21–0.70) *** | 0.80 (0.50–1.29) |
| Model 2 | 0.99 (0.78–1.26) | 1.00 (ref) | 0.37 (0.20–0.67) *** | 0.84 (0.51–1.39) |
| Breast cancer |  |  |  |  |
| No. of events/subjects | 135/6109 | 66/2043 | 42/2030 | 27/2036 |
| Univariate | 0.67 (0.58–0.78) *** | 1.00 (ref) | 0.64 (0.44–0.95) * | 0.42 (0.27–0.65) *** |
| Model 1 | 0.88 (0.73–1.06) | 1.00 (ref) | 0.92 (0.61–1.38) | 0.98 (0.60–1.61) |
| Model 2 | 0.86 (0.71–1.05) | 1.00 (ref) | 0.91 (0.60–1.38) | 0.98 (0.58–1.63) |
| GI cancer | | | | |
| No. of events/subjects | 162/6109 | 32/2043 | 68/2030 | 62/2036 |
| Univariate | 1.27 (1.08–1.49) *** | 1.00 (ref) | 2.17 (1.43–3.31) *** | 1.99 (1.30–3.04) *** |
| Model 1 | 0.92 (0.78–1.10) | 1.00 (ref) | 1.37 (0.89–2.10) | 0.98 (0.63–1.53) |
| Model 2 | 0.92 (0.76–1.10) | 1.00 (ref) | 1.35 (0.88–2.08) | 0.98 (0.61–1.55) |
| Prostate cancer |  |  |  |  |
| No. of events/subjects | 94/6109 | 12/2043 | 34/2030 | 48/2036 |
| Univariate | 1.77 (1.42–2.22) *** | 1.00 (ref) | 2.89 (1.50–5.59) *** | 4.12 (2.19–7.75) *** |
| Model 1 | 1.06 (0.83–1.34) | 1.00 (ref) | 1.32 (0.68–2.56) | 1.20 (0.63–2.26) |
| Model 2 | 1.13 (0.87–1.46) | 1.00 (ref) | 1.46 (0.75–2.85) | 1.33 (0.69–2.55) |
| Hematological cancer | | | | |
| No. of events/subjects | 70/6109 | 24/2043 | 19/2030 | 27/2036 |
| Univariate | 1.11 (0.88–1.40) | 1.00 (ref) | 0.81 (0.44–1.47) | 1.16 (0.67–2.00) |
| Model 1 | 0.87 (0.67–1.13) | 1.00 (ref) | 0.56 (0.30–1.03) | 0.68 (0.38–1.22) |
| Model 2 | 0.84 (0.64–1.10) | 1.00 (ref) | 0.55 (0.29–1.02) | 0.62 (0.33–1.15) |
| Urothelial cell cancer | | | | |
| No. of events/subjects | 82/6109 | 19/2043 | 17/2030 | 46/2036 |
| Univariate | 1.62 (1.28–2.05) *** | 1.00 (ref) | 0.91 (0.47–1.75) | 2.49 (1.46–4.26) *** |
| Model 1 | 1.11 (0.86–1.43) | 1.00 (ref) | 0.50 (0.26–0.97) * | 1.00 (0.58–1.73) |
| Model 2 | 1.16 (0.88–1.52) | 1.00 (ref) | 0.49 (0.25–0.96) * | 1.07 (0.60–1.91) |

HRs and 95% CIs were derived from Cox proportional hazards regression models.

Model 1: adjusted for age, sex and race.

Model 2: as model 1+ adjusted for baseline eGFR, BMI, smoking, alcohol, educational level and type 2 diabetes, protein, diabetic medication, lipid lowering drugs, serum protein, hypertension, systolic blood pressure, UAE, hs-CRP and total cholesterol.

Abbreviations: GI, gastrointestinal; BMI, body mass index; eGFR, estimated glomerular filtration rate; UAE, urinary albumin excretion; hs-CRP, high sensitivity c-reactive protein.

*p* < 0.05: *; *p* < 0.01: **; *p* < 0.0625: ***

| Table S5\| Association of TSAT with cancer development | | | | |
| --- | --- | --- | --- | --- |
|  |  | **Tertiles of TSAT (%)** | | |
|  | **TSAT as continuous variable** | **T1: < 20.7** | **T2: 20.7–27.8** | **T3: > 27.8** |
| Cancer | | | | |
| No. of events/subjects | 645/6109 | 230/2078 | 204/2000 | 211/2031 |
| Univariate | 1.00 (0.99–1.01) | 1.00 (ref) | 0.93 (0.77–1.12) | 0.94 (0.78–1.13) |
| Model 1 | 0.99 (0.98–1.00) | 1.00 (ref) | 0.85 (0.70–1.02) | 0.83 (0.69–1.00) |
| Model 2 | 0.99 (0.99–1.00) | 1.00 (ref) | 0.88 (0.73–1.07) | 0.85 (0.70–1.04) |
| Skin cancer | | | | |
| No. of events/subjects | 445/6109 | 154/2078 | 138/2000 | 153/2031 |
| Univariate | 1.00 (0.99–1.01) | 1.00 (ref) | 0.92 (0.73–1.15) | 1.01 (0.81–1.26) |
| Model 1 | 1.00 (0.98–1.01) | 1.00 (ref) | 0.82 (0.65–1.03) | 0.87 (0.70–1.10) |
| Model 2 | 0.99 (0.98–1.00) | 1.00 (ref) | 0.80 (0.63–1.01) | 0.85 (0.68–1.07) |
| Lung cancer | | | | |
| No. of events/subjects | 108/6109 | 37/2078 | 37/2000 | 34/2031 |
| Univariate | 1.00 (0.98–1.02) | 1.00 (ref) | 1.04 (0.66–1.64) | 0.94 (0.59–1.5) |
| Model 1 | 0.98 (0.96–1.01) | 1.00 (ref) | 0.89 (0.56–1.41) | 0.73 (0.46–1.17) |
| Model 2 | 0.99 (0.96–1.01) | 1.00 (ref) | 0.93 (0.59–1.48) | 0.77 (0.47–1.25) |
| Kidney cancer | | | | |
| No. of events/subjects | 99/6109 | 28/2078 | 38/2000 | 33/2031 |
| Univariate | 1.00 (0.98–1.02) | 1.00 (ref) | 1.42 (0.87–2.31) | 1.21 (0.73–2.00) |
| Model 1 | 0.99 (0.97–1.01) | 1.00 (ref) | 1.17 (0.72–1.90) | 0.86 (0.52–1.42) |
| Model 2 | 0.99 (0.97–1.01) | 1.00 (ref) | 1.23 (0.75–2.02) | 0.90 (0.53–1.53) |
| Breast cancer | | | | |
| No. of events/subjects | 135/6109 | 59/2078 | 43/2000 | 33/2031 |
| Univariate | 0.98 (0.96–0.99) * | 1.00 (ref) | 0.76 (0.51–1.12) | 0.57 (0.37–0.88) * |
| Model 1 | 0.99 (0.98–1.01) | 1.00 (ref) | 0.91 (0.61–1.35) | 0.85 (0.55–1.30) |
| Model 2 | 0.99 (0.97–1.01) | 1.00 (ref) | 0.89 (0.60–1.33) | 0.81 (0.52–1.25) |
| GI cancer | | | | |
| No. of events/subjects | 162/6109 | 62/2078 | 48/2000 | 52/2031 |
| Univariate | 1.00 (0.98–1.01) | 1.00 (ref) | 0.80 (0.55–1.17) | 0.86 (0.59–1.24) |
| Model 1 | 0.99 (0.97–1.00) | 1.00 (ref) | 0.68 (0.47–0.99) * | 0.66 (0.45–0.96) * |
| Model 2 | 0.98 (0.97–1.00) | 1.00 (ref) | 0.66 (0.45–0.97) * | 0.61 (0.42–0.90) * |
| Prostate cancer | | | | |
| No. of events/subjects | 94/6109 | 23/2078 | 34/2000 | 37/2031 |
| Univariate | 1.02 (1.00–1.04) | 1.00 (ref) | 1.53 (0.90–2.60) | 1.65 (0.98–2.77) |
| Model 1 | 1.00 (0.98–1.02) | 1.00 (ref) | 1.16 (0.68–1.97) | 0.97 (0.58–1.63) |
| Model 2 | 1.00 (0.97–1.02) | 1.00 (ref) | 1.19 (0.70–2.03) | 0.91 (0.53–1.55) |
| Hematological cancer | | | | |
| No. of events/subjects | 70/6109 | 30/2078 | 23/2000 | 17/2031 |
| Univariate | 0.98 (0.95–1.01) | 1.00 (ref) | 0.75 (0.43–1.30) | 0.56 (0.31–1.02) |
| Model 1 | 0.97 (0.94–1.00) * | 1.00 (ref) | 0.66 (0.38–1.15) | 0.48 (0.26–0.87) * |
| Model 2 | 0.97 (0.95–1.00) | 1.00 (ref) | 0.71 (0.41–1.24) | 0.51 (0.28–0.95) * |
| Urothelial cell cancer |  |  |  |  |
| No. of events/subjects | 82/6109 | 21/2078 | 34/2000 | 27/2031 |
| Univariate | 1.01 (0.99–1.03) | 1.00 (ref) | 1.69 (0.98–2.91) | 1.31 (0.74–2.32) |
| Model 1 | 1.00 (0.97–1.02) | 1.00 (ref) | 1.38 (0.80–2.37) | 0.90 (0.51–1.61) |
| Model 2 | 1.00 (0.97–1.02) | 1.00 (ref) | 1.42 (0.81–2.47) | 0.93 (0.51–1.69) |

HRs and 95% CIs were derived from Cox proportional hazards regression models.

Model 1: adjusted for age, sex and race.

Model 2: as model 1+ adjusted for baseline eGFR, BMI, smoking, alcohol, educational level and type 2 diabetes, protein, diabetic medication, lipid lowering drugs, serum protein, hypertension, systolic blood pressure, UAE, hs-CRP and total cholesterol.

Abbreviations: TSAT, transferrin saturation; sTfR, soluble transferrin receptor; GI, gastrointestinal; BMI, body mass index; eGFR, estimated glomerular filtration rate; UAE, urinary albumin excretion; hs-CRP, high sensitivity c-reactive protein.

*p* < 0.05: *; *p* < 0.01: **; *p* < 0.0625: ***

**Table S6|** Association of sTfR with cancer development

|  |  | Tertiles of sTfR (mg/L) | | |
| --- | --- | --- | --- | --- |
|  | **sTfR as continuous variable** | **T1: <2.2** | **T2: 2.2–2.82** | **T3: >2.82** |
| Overall cancer |  |  |  |  |
| No. of events/subjects | 645/6109 | 214/2038 | 189/2057 | 242/2014 |
| Univariate | 1.32 (1.02–1.72) * | 1.00 (ref) | 0.98 (0.77–1.25) | 1.18 (0.97–1.44) |
| Model 1 | 1.21 (0.91–1.60) | 1.00 (ref) | 0.88 (0.69–1.13) | 1.04 (0.85–1.27) |
| Model 2 | 1.35 (1.01–1.80) * | 1.00 (ref) | 0.95 (0.74–1.22) | 1.15 (0.93–1.42) |
| Skin cancer |  |  |  |  |
| No. of events/subjects | 445/6109 | 130/2038 | 160/2057 | 155/2014 |
| Univariate | 1.3 (0.95–1.78) | 1.00 (ref) | 1.26 (1.00–1.60) | 1.29 (1.01–1.65) * |
| Model 1 | 1.2 (0.84–1.71) | 1.00 (ref) | 1.14 (0.90–1.45) | 1.14 (0.88–1.46) |
| Model 2 | 1.26 (0.88–1.8) | 1.00 (ref) | 1.17 (0.92–1.49) | 1.18 (0.91–1.53) |
| Lung cancer |  |  |  |  |
| No. of events/subjects | 108/6109 | 37/2038 | 39/2057 | 32/2014 |
| Univariate | 1.01 (0.52–1.96) | 1.00 (ref) | 1.38 (0.76–2.51) | 1.06 (0.60–1.87) |
| Model 1 | 0.74 (0.34–1.58) | 1.00 (ref) | 1.16 (0.64–2.11) | 0.82 (0.45–1.47) |
| Model 2 | 1.01 (0.46–2.20) | 1.00 (ref) | 1.40 (0.76–2.58) | 1.07 (0.58–1.98) |
| Kidney cancer |  |  |  |  |
| No. of events/subjects | 99/6109 | 33/2038 | 31/2057 | 35/2014 |
| Univariate | 1.14 (0.54–2.41) | 1.00 (ref) | 0.96 (0.58–1.58) | 0.98 (0.57–1.68) |
| Model 1 | 0.89 (0.38–2.09) | 1.00 (ref) | 0.81 (0.49–1.34) | 0.77 (0.45–1.32) |
| Model 2 | 1.03 (0.44–2.43) | 1.00 (ref) | 0.89 (0.54–1.48) | 0.86 (0.49–1.52) |
| Breast cancer |  |  |  |  |
| No. of events/subjects | 135/6109 | 45/2038 | 47/2057 | 43/2014 |
| Univariate | 0.81 (0.46–1.45) | 1.00 (ref) | 1.12 (0.72–1.75) | 0.99 (0.64–1.55) |
| Model 1 | 0.86 (0.50–1.49) | 1.00 (ref) | 1.14 (0.73–1.80) | 1.03 (0.66–1.62) |
| Model 2 | 1.04 (0.59–1.85) | 1.00 (ref) | 1.28 (0.81–2.03) | 1.23 (0.76–1.97) |
| GI cancer |  |  |  |  |
| No. of events/subjects | 162/6109 | 65/2038 | 44/2057 | 53/2014 |
| Univariate | 0.98 (0.58–1.64) | 1.00 (ref) | 0.72 (0.47–1.10) | 0.87 (0.59–1.29) |
| Model 1 | 0.79 (0.44–1.41) | 1.00 (ref) | 0.63 (0.41–0.96) * | 0.72 (0.48–1.07) |
| Model 2 | 1.00 (0.55–1.82) | 1.00 (ref) | 0.70 (0.46–1.06) | 0.85 (0.56–1.29) |
| Prostate cancer | | | | |
| No. of events/subjects | 94/6109 | 24/2038 | 26/2057 | 44/2014 |
| Univariate | 2.11 (1.20–3.72) * | 1.00 (ref) | 1.15 (0.61–2.18) | 1.97 (1.15–3.39) * |
| Model 1 | 1.71 (0.85–3.47) | 1.00 (ref) | 0.92 (0.48–1.75) | 1.38 (0.79–2.38) |
| Model 2 | 1.63 (0.79–3.36) | 1.00 (ref) | 0.86 (0.44–1.66) | 1.33 (0.75–2.36) |
| Hematological cancer | | | | |
| No. of events/subjects | 70/6109 | 15/2038 | 16/2057 | 39/2014 |
| Univariate | 2.28 (1.14–4.59) * | 1.00 (ref) | 1.29 (0.63–2.65) | 2.35 (1.24–4.46) * |
| Model 1 | 2.27 (1.07–4.84) * | 1.00 (ref) | 1.16 (0.56–2.38) | 2.05 (1.07–3.91) * |
| Model 2 | 1.96 (0.89–4.34) | 1.00 (ref) | 1.13 (0.55–2.35) | 1.92 (0.97–3.78) |
| Urothelial cell cancer | | | | |
| No. of events/subjects | 82/6109 | 31/2038 | 25/2057 | 26/2014 |
| Univariate | 0.80 (0.33–1.91) | 1.00 (ref) | 0.81 (0.47–1.41) | 0.78 (0.43–1.43) |
| Model 1 | 0.52 (0.19–1.40) | 1.00 (ref) | 0.67 (0.38–1.15) | 0.58 (0.32–1.05) |
| Model 2 | 0.62 (0.22–1.74) | 1.00 (ref) | 0.72 (0.41–1.26) | 0.65 (0.35–1.21) |

HRs and 95% CIs were derived from Cox proportional hazards regression models.

Model 1: adjusted for age, sex and race.

Model 2: as model 1+ adjusted for baseline eGFR, BMI, smoking, alcohol, educational level and type 2 diabetes, protein, diabetic medication, lipid lowering drugs, serum protein, hypertension, systolic blood pressure, UAE, hs-CRP and total cholesterol.

Abbreviations: sTfR, soluble transferrin receptor; GI, gastrointestinal; BMI, body mass index; eGFR, estimated glomerular filtration rate; UAE, urinary albumin excretion; hs-CRP, high sensitivity c-reactive protein.

*p* < 0.05: *; *p* < 0.01: **; *p* < 0.0625: ***

**Table S7|** Association of hepcidin with cancer development

|  |  | Tertiles of hepcidin (nM) | | |  |
| --- | --- | --- | --- | --- | --- |
|  | **Hepcidin as continuous variable** | **T1: < 2.1** | **T2: 2.1–4.1** | **T3: >4.1** |  |
| Overall cancer | | | | | |
| No. of events/subjects | 645/6109 | 208/2036 | 189/2035 | 248/2038 |  |
| Univariate | 1.09 (1.00–1.19) | 1.00 (ref) | 0.92 (0.75–1.12) | 1.21 (1.01–1.46) * |  |
| Model 1 | 0.89 (0.81–0.97) * | 1.00 (ref) | 0.69 (0.56–0.84) *** | 0.80 (0.66–0.96) * |  |
| Model 2 | 0.88 (0.80–0.96) ** | 1.00 (ref) | 0.69 (0.56–0.84) *** | 0.78 (0.64–0.95) * |  |
| Skin cancer | | | | | |
| No. of events/subjects | 445/6109 | 110/2036 | 163/2035 | 172/2038 |  |
| Univariate | 1.25 (1.12–1.39) *** | 1.00 (ref) | 1.52 (1.19–1.94) *** | 1.64 (1.29–2.09) *** |  |
| Model 1 | 1.02 (0.91–1.14) | 1.00 (ref) | 1.14 (0.89–1.46) | 1.08 (0.84–1.38) |  |
| Model 2 | 1.06 (0.94–1.19) | 1.00 (ref) | 1.19 (0.92–1.52) | 1.15 (0.89–1.49) |  |
| Lung cancer | | | | | |
| No. of events/subjects | 108/6109 | 26/2036 | 29/2035 | 53/2038 |  |
| Univariate | 1.43 (1.14–1.79) *** | 1.00 (ref) | 1.16 (0.69–1.97) | 2.00 (1.24–3.23) *** |  |
| Model 1 | 1.09 (0.86–1.38) | 1.00 (ref) | 0.79 (0.47–1.34) | 1.12 (0.69–1.82) |  |
| Model 2 | 0.97 (0.76–1.24) | 1.00 (ref) | 0.71 (0.42–1.21) | 0.88 (0.53–1.47) |  |
| Kidney cancer | | | | | |
| No. of events/subjects | 99/6109 | 21/2036 | 31/2035 | 47/2038 |  |
| Univariate | 1.50 (1.18–1.90) *** | 1.00 (ref) | 1.53 (0.87–2.69) | 2.35 (1.40–3.96) *** |  |
| Model 1 | 1.13 (0.88–1.45) | 1.00 (ref) | 1.00 (0.57–1.76) | 1.27 (0.75–2.15) |  |
| Model 2 | 1.11 (0.86–1.43) | 1.00 (ref) | 0.95 (0.53–1.69) | 1.23 (0.71–2.14) |  |
| Breast cancer | | | | | |
| No. of events/subjects | 135/6109 | 61/2036 | 39/2035 | 35/2038 |  |
| Univariate | 0.72 (0.60–0.85) *** | 1.00 (ref) | 0.64 (0.43–0.95) * | 0.58 (0.39–0.89) * |  |
| Model 1 | 0.84 (0.69–1.01) | 1.00 (ref) | 0.82 (0.54–1.25) | 0.84 (0.53–1.33) |  |
| Model 2 | 0.82 (0.68–1.00) | 1.00 (ref) | 0.82 (0.53–1.25) | 0.85 (0.53–1.37) |  |
| GI cancer | | | | | |
| No. of events/subjects | 162/6109 | 52/2036 | 47/2035 | 63/2038 |  |
| Univariate | 1.08 (0.91–1.28) | 1.00 (ref) | 0.90 (0.61–1.34) | 1.24 (0.86–1.79) |  |
| Model 1 | 0.82 (0.69–0.98) * | 1.00 (ref) | 0.62 (0.41–0.92) * | 0.71 (0.49–1.03) |  |
| Model 2 | 0.82 (0.68–0.98) * | 1.00 (ref) | 0.63 (0.42–0.95) * | 0.72 (0.49–1.06) |  |
| Prostate cancer | | | | | |
| No. of events/subjects | 94/6109 | 23/2036 | 28/2035 | 43/2038 |  |
| Univariate | 1.41 (1.11–1.79) ** | 1.00 (ref) | 1.15 (0.66–2.00) | 1.85 (1.12–3.06) * |  |
| Model 1 | 0.96 (0.75–1.24) | 1.00 (ref) | 0.68 (0.39–1.19) | 0.83 (0.50–1.38) |  |
| Model 2 | 0.99 (0.77–1.29) | 1.00 (ref) | 0.72 (0.41–1.27) | 0.91 (0.53–1.55) |  |
| Hematological cancer |  |  |  |  |  |
| No. of events/subjects | 70/6109 | 21/2036 | 17/2035 | 32/2038 |  |
| Univariate | 1.44 (1.09–1.91) * | 1.00 (ref) | 0.82 (0.43–1.55) | 1.57 (0.90–2.72) |  |
| Model 1 | 1.18 (0.88–1.58) | 1.00 (ref) | 0.60 (0.32–1.15) | 1.01 (0.57–1.77) |  |
| Model 2 | 1.11 (0.83–1.49) | 1.00 (ref) | 0.62 (0.32–1.20) | 0.94 (0.52–1.71) |  |
| Urothelial cell cancer | | | | | |
| No. of events/subjects | 82/6109 | 14/2036 | 29/2035 | 39/2038 |  |
| Univariate | 1.65 (1.26–2.15) *** | 1.00 (ref) | 2.08 (1.10–3.95) * | 2.87 (1.56–5.28) *** |  |
| Model 1 | 1.23 (0.93–1.63) | 1.00 (ref) | 1.33 (0.70–2.53) | 1.49 (0.81–2.75) |  |
| Model 2 | 1.21 (0.90–1.61) | 1.00 (ref) | 1.27 (0.66–2.43) | 1.44 (0.76–2.71) |  |

HRs and 95% CIs were derived from Cox proportional hazards regression models.

Model 1: adjusted for age, sex and race.

Model 2: as model 1+ adjusted for baseline eGFR, BMI, smoking, alcohol, educational level and type 2 diabetes, protein, diabetic medication, lipid lowering drugs, serum protein, hypertension, systolic blood pressure, UAE, hs-CRP and total cholesterol.

Abbreviations: TSAT, transferrin saturation; GI, gastrointestinal; BMI, body mass index; eGFR, estimated glomerular filtration rate; UAE, urinary albumin excretion; hs-CRP, high sensitivity c-reactive protein.

*p* < 0.05: *; *p* < 0.01: **; *p* < 0.0625: ***

**Table S8|** Association of EPO with cancer development

|  |  |  | **Tertiles of EPO** |  |
| --- | --- | --- | --- | --- |
|  | **EPO as continuous variable** | **T1: <6.50** | **T2: 6.50–9.22** | **T3: >9.22** |
| **Overall cancer** | | | | |
| **No. of events/subjects** | 645/6109 | 179/2042 | 224/2037 | 242/2030 |
| **Univariate** | 1.36 (1.17–1.58) *** | 1.00 (ref) | 1.27 (1.04–1.55) * | 1.41 (1.16–1.71) *** |
| **Model 1** | 1.29 (1.10–1.51) *** | 1.00 (ref) | 1.21 (0.99–1.47) | 1.23 (1.01–1.50) * |
| **Model 2** | 1.26 (1.07–1.47) *** | 1.00 (ref) | 1.21 (0.99–1.48) | 1.21 (0.99–1.48) |
| **Skin cancer** | | | | |
| **No. of events/subjects** | 445/6109 | 137/2042 | 154/2037 | 154/2030 |
| **Univariate** | 1.02 (0.84–1.23) | 1.00 (ref) | 1.13 (0.90–1.42) | 1.13 (0.89–1.42) |
| **Model 1** | 0.94 (0.77–1.14) | 1.00 (ref) | 1.07 (0.85–1.35) | 0.97 (0.77–1.23) |
| **Model 2** | 0.91 (0.75–1.12) | 1.00 (ref) | 1.07 (0.85–1.35) | 0.95 (0.74–1.20) |
| **Lung cancer** | | | | |
| **No. of events/subjects** | 108/6109 | 37/2042 | 32/2037 | 39/2030 |
| **Univariate** | 1.01 (0.69–1.48) | 1.00 (ref) | 0.90 (0.56–1.44) | 1.09 (0.69–1.73) |
| **Model 1** | 0.89 (0.60–1.33) | 1.00 (ref) | 0.83 (0.51–1.33) | 0.90 (0.56–1.42) |
| **Model 2** | 0.99 (0.66–1.49) | 1.00 (ref) | 0.89 (0.55–1.44) | 0.98 (0.61–1.59) |
| **Kidney cancer** | | | | |
| **No. of events/subjects** | 99/6109 | 26/2042 | 40/2037 | 33/2030 |
| **Univariate** | 1.26 (0.87–1.84) | 1.00 (ref) | 1.59 (0.96–2.62) | 1.35 (0.80–2.27) |
| **Model 1** | 1.21 (0.80–1.82) | 1.00 (ref) | 1.51 (0.92–2.49) | 1.15 (0.68–1.95) |
| **Model 2** | 1.14 (0.75–1.75) | 1.00 (ref) | 1.51 (0.91–2.50) | 1.08 (0.63–1.86) |
| **Breast cancer** | | | | |
| **No. of events/subjects** | 135/6109 | 46/2042 | 48/2037 | 41/2030 |
| **Univariate** | 1.00 (0.71–1.40) | 1.00 (ref) | 1.06 (0.71–1.60) | 0.89 (0.58–1.36) |
| **Model 1** | 0.85 (0.61–1.19) | 1.00 (ref) | 0.99 (0.65–1.48) | 0.74 (0.48–1.13) |
| **Model 2** | 0.84 (0.59–1.19) | 1.00 (ref) | 0.96 (0.64–1.45) | 0.72 (0.46–1.12) |
| **Gastrointestinal cancer** | | | | |
| **No. of events/subjects** | 162/6109 | 49/2042 | 51/2037 | 62/2030 |
| **Univariate** | 1.18 (0.86–1.60) | 1.00 (ref) | 1.07 (0.72–1.59) | 1.31 (0.90–1.91) |
| **Model 1** | 1.11 (0.80–1.54) | 1.00 (ref) | 1.01 (0.68–1.50) | 1.13 (0.77–1.65) |
| **Model 2** | 1.15 (0.83–1.61) | 1.00 (ref) | 1.05 (0.71–1.57) | 1.21 (0.82–1.79) |
| **Prostate** | | | | |
| **No. of events/subjects** | 94/6109 | 23/2042 | 35/2037 | 36/2030 |
| **Univariate** | 1.42 (0.98–2.05) | 1.00 (ref) | 1.58 (0.93–2.69) | 1.67 (0.98–2.84) |
| **Model 1** | 1.36 (0.9–2.05) | 1.00 (ref) | 1.50 (0.88–2.55) | 1.40 (0.82–2.39) |
| **Model 2** | 1.30 (0.85–2.00) | 1.00 (ref) | 1.46 (0.85–2.52) | 1.37 (0.78–2.38) |
| **Hematological cancer** | | | | |
| **No. of events/subjects** | 70/6109 | 18/2042 | 27/2037 | 25/2030 |
| **Univariate** | 1.62 (1.09–2.42) * | 1.00 (ref) | 1.47 (0.80–2.68) | 1.42 (0.77–2.61) |
| **Model 1** | 1.59 (1.02–2.47) * | 1.00 (ref) | 1.37 (0.75–2.51) | 1.21 (0.66–2.23) |
| **Model 2** | 1.43 (0.90–2.27) | 1.00 (ref) | 1.37 (0.74–2.51) | 1.05 (0.56–1.99) |
| **Urothelial cell cancer** | | | | |
| **No. of events/subjects** | 82/6109 | 24/2042 | 31/2037 | 27/2030 |
| **Univariate** | 1.07 (0.69–1.65) | 1.00 (ref) | 1.34 (0.78–2.29) | 1.20 (0.69–2.10) |
| **Model 1** | 0.96 (0.61–1.53) | 1.00 (ref) | 1.25 (0.73–2.15) | 0.98 (0.56–1.72) |
| **Model 2** | 0.97 (0.60–1.57) | 1.00 (ref) | 1.29 (0.75–2.24) | 0.99 (0.56–1.78) |

HRs and 95% CIs were derived from Cox proportional hazards regression models.

Model 1: adjusted for age, sex and race.

Model 2: as model 1+ adjusted for baseline eGFR, BMI, smoking, alcohol, educational level and type 2 diabetes, protein, diabetic medication, lipid lowering drugs, serum protein, hypertension, systolic blood pressure, UAE, hs-CRP and total cholesterol.

Abbreviations: TSAT, transferrin saturation; EPO, erythropoietin; GI, gastrointestinal; BMI, body mass index; eGFR, estimated glomerular filtration rate; UAE, urinary albumin excretion; hs-CRP, high sensitivity c-reactive protein.

*p* < 0.05: *; *p* < 0.01: **; *p* < 0.0625: ***

**Table S9|** Association of ferritin with cancer development for cancers diagnosed 1 year post blood sampling

|  | | | **Tertiles of ferritin (µg/L)** | | |
| --- | --- | --- | --- | --- | --- |
|  | | **Ferritin as**  **continuous variable** | **T1: < 61** | **T2: 61-139** | **T3: >139** |
| **Overall cancer** | |  |  |  |  |
| No. of events/subjects | | 608/6053 | 183/2029 | 196/2011 | 229/2013 |
| Univariate | | 1.12 (1.03–1.21) ** | 1.00 (ref) | 1.09 (0.89–1.33) | 1.28 (1.05–1.55) * |
| Model 1 | | 0.90 (0.83–0.99) * | 1.00 (ref) | 0.79 (0.64–0.97) * | 0.80 (0.65–0.98) * |
| Model 2 | | 0.89 (0.81–0.98) * | 1.00 (ref) | 0.77 (0.62–0.95) * | 0.76 (0.61–0.94) * |
| **Skin cancer** | | |  |  |  |
| No. of events/subjects | | 426/6053 | 105/2029 | 157/2011 | 164/2013 |
| Univariate | | 1.21 (1.10–1.34) *** | 1.00 (ref) | 1.54 (1.20–1.97) *** | 1.64 (1.28–2.09) *** |
| Model 1 | | 0.99 (0.89–1.10) | 1.00 (ref) | 1.09 (0.85–1.41) | 1.03 (0.79–1.33) |
| Model 2 | | 1.00 (0.90–1.12) | 1.00 (ref) | 1.13 (0.87–1.46) | 1.11 (0.84–1.45) |
| **Lung cancer** | | |  |  |  |
| No. of events/subjects | | 101/6053 | 26/2029 | 27/2011 | 48/2013 |
| Univariate | | 1.55 (1.26–1.92) *** | 1.00 (ref) | 1.06 (0.62–1.81) | 1.89 (1.17–3.05) * |
| Model 1 | | 1.16 (0.92–1.46) | 1.00 (ref) | 0.65 (0.38–1.12) | 0.92 (0.56–1.51) |
| Model 2 | | 1.12 (0.88–1.42) | 1.00 (ref) | 0.56 (0.32–0.98) * | 0.71 (0.42–1.20) |
| **Kidney cancer** | | |  |  |  |
| No. of events/subjects | | 95/6053 | 26/2029 | 19/2011 | 50/2013 |
| Univariate | | 1.43 (1.15–1.77) *** | 1.00 (ref) | 0.74 (0.41–1.34) | 1.99 (1.24–3.19) ** |
| Model 1 | | 0.99 (0.79–1.25) | 1.00 (ref) | 0.42 (0.23–0.76) ** | 0.84 (0.51–1.38) |
| Model 2 | | 0.96 (0.75–1.22) | 1.00 (ref) | 0.40 (0.22–0.74) *** | 0.88 (0.52–1.48) |
| **Breast cancer** | | |  |  |  |
| No. of events/subjects | | 129/6053 | 65/2029 | 39/2011 | 25/2013 |
| Univariate | | 0.66 (0.56–0.77) *** | 1.00 (ref) | 0.61 (0.41–0.90) * | 0.39 (0.25–0.62) *** |
| Model 1 | | 0.86 (0.71–1.04) | 1.00 (ref) | 0.88 (0.58–1.33) | 0.94 (0.57–1.57) |
| Model 2 | | 0.85 (0.70–1.03) | 1.00 (ref) | 0.87 (0.57–1.33) | 0.93 (0.55–1.58) |
| **GI cancer** | | | |  |  |
| No. of events/subjects | | 154/6053 | 29/2029 | 65/2011 | 60/2013 |
| Univariate | | 1.31 (1.11–1.55) *** | 1.00 (ref) | 2.29 (1.48–3.55) *** | 2.13 (1.37–3.31) *** |
| Model 1 | | 0.94 (0.79–1.13) | 1.00 (ref) | 1.41 (0.90–2.20) | 1.01 (0.64–1.60) |
| Model 2 | | 0.92 (0.76–1.11) | 1.00 (ref) | 1.38 (0.88–2.16) | 0.99 (0.62–1.60) |
| **Prostate cancer** | | |  |  |  |
| No. of events/subjects | | 89/6053 | 12/2029 | 34/2011 | 43/2013 |
| Univariate | | 1.69 (1.34–2.13) *** | 1.00 (ref) | 2.90 (1.50–5.59) *** | 3.69 (1.95–7.00) *** |
| Model 1 | | 0.99 (0.78–1.27) | 1.00 (ref) | 1.30 (0.67–2.51) | 1.05 (0.55–1.99) |
| Model 2 | | 1.03 (0.79–1.33) | 1.00 (ref) | 1.43 (0.73–2.78) | 1.16 (0.60–2.24) |
| **Hematological cancer** | | | |  |  |
| No. of events/subjects | 64/6053 | | 23/2029 | 18/2011 | 23/2013 |
| Univariate | 1.06 (0.83–1.35) | | 1.00 (ref) | 0.80 (0.43–1.48) | 1.03 (0.58–1.83) |
| Model 1 | | 0.83 (0.63–1.08) | 1.00 (ref) | 0.55 (0.29–1.03) | 0.59 (0.32–1.10) |
| Model 2 | | 0.78 (0.59–1.03) | 1.00 (ref) | 0.52 (0.28–1.00) | 0.51 (0.27–0.98) |
| **Urothelial cell cancer** | | | |  |  |
| No. of events/subjects | 78/6053 | | 17/2029 | 17/2011 | 44/2013 |
| Univariate | 1.67 (1.31–2.13) *** | | 1.00 (ref) | 1.02 (0.52–2.00) | 2.67 (1.53–4.68) *** |
| Model 1 | | 1.15 (0.89–1.50) | 1.00 (ref) | 0.56 (0.29–1.11) | 1.09 (0.61–1.93) |
| Model 2 | | 1.20 (0.90–1.60) | 1.00 (ref) | 0.55 (0.28–1.10) | 1.15 (0.63–2.11) |

HRs and 95% CIs were derived from Cox proportional hazards regression models.
Model 1: adjusted for age, sex and race.
Model 2: as model 1+ adjusted for baseline eGFR, BMI, smoking, alcohol, educational level and type 2 diabetes, protein, diabetic medication, lipid lowering drugs, serum protein, hypertension, systolic blood pressure, UAE, hs-CRP and total cholesterol. Abbreviations: BMI, body mass index; eGFR, estimated glomerular filtration rate; UAE, urinary albumin excretion; hs-CRP, high sensitivity c-reactive protein
*p* < 0.05: *; *p* < 0.01: **; *p* < 0.0625: ***

**Table S10|** Association of TSAT with cancer development for cancers diagnosed 1 year post blood sampling

|  | | **Tertiles of TSAT (%)** | | |
| --- | --- | --- | --- | --- |
|  | **TSAT as**  **continuous variable** | **T1: < 20.7** | **T2: 20.7-27.8** | **T3: >27.8** |
| **Overall cancer** |  |  |  |  |
| No. of events/subjects | 608/6053 | 213/2050 | 195/1985 | 200/2018 |
| Univariate | 1.00 (0.99–1.01) | 1.00 (ref) | 0.95 (0.79–1.16) | 0.96 (0.79–1.16) |
| Model 1 | 0.99 (0.98–1.00) | 1.00 (ref) | 0.87 (0.72–1.06) | 0.85 (0.70–1.03) |
| Model 2 | 0.99 (0.99–1.00) | 1.00 (ref) | 0.90 (0.74–1.10) | 0.87 (0.71–1.06) |
| **Skin cancer** |  |  |  |  |
| No. of events/subjects | 426/6053 | 143/2050 | 132/1985 | 151/2018 |
| Univariate | 1.00 (0.99–1.01) | 1.00 (ref) | 0.94 (0.74–1.19) | 1.07 (0.85–1.35) |
| Model 1 | 1.00 (0.99–1.01) | 1.00 (ref) | 0.84 (0.66–1.07) | 0.93 (0.74–1.18) |
| Model 2 | 1.00 (0.99–1.01) | 1.00 (ref) | 0.82 (0.65–1.04) | 0.91 (0.71–1.15) |
| **Lung cancer** |  |  |  |  |
| No. of events/subjects | 101/6053 | 34/2050 | 35/1985 | 32/2018 |
| Univariate | 1.00 (0.98–1.02) | 1.00 (ref) | 1.07 (0.66–1.71) | 0.96 (0.59–1.56) |
| Model 1 | 0.98 (0.96–1.01) | 1.00 (ref) | 0.92 (0.57–1.47) | 0.75 (0.46–1.23) |
| Model 2 | 0.99 (0.96–1.01) | 1.00 (ref) | 0.93 (0.58–1.51) | 0.77 (0.46–1.27) |
| **Kidney cancer** |  |  |  |  |
| No. of events/subjects | 95/6053 | 26/2050 | 37/1985 | 32/2018 |
| Univariate | 1.01 (0.98–1.03) | 1.00 (ref) | 1.48 (0.90–2.45) | 1.26 (0.75–2.11) |
| Model 1 | 0.99 (0.97–1.01) | 1.00 (ref) | 1.23 (0.74–2.03) | 0.90 (0.53–1.52) |
| Model 2 | 0.99 (0.97–1.02) | 1.00 (ref) | 1.32 (0.79–2.20) | 0.97 (0.57–1.67) |
| **Breast cancer** |  |  |  |  |
| No. of events/subjects | 129/6053 | 56/2050 | 41/1985 | 32/2018 |
| Univariate | 0.98 (0.96–1.00) * | 1.00 (ref) | 0.76 (0.51–1.14) | 0.58 (0.38–0.90) * |
| Model 1 | 1.00 (0.98–1.01) | 1.00 (ref) | 0.91 (0.61–1.37) | 0.87 (0.56–1.34) |
| Model 2 | 0.99 (0.97–1.01) | 1.00 (ref) | 0.90 (0.59–1.35) | 0.82 (0.53–1.28) |
| **GI cancer** |  |  |  |  |
| No. of events/subjects | 154/6053 | 56/2050 | 47/1985 | 51/2018 |
| Univariate | 1.00 (0.99–1.02) | 1.00 (ref) | 0.87 (0.59–1.28) | 0.93 (0.63–1.36) |
| Model 1 | 0.99 (0.97–1.01) | 1.00 (ref) | 0.73 (0.49–1.07) | 0.70 (0.48–1.03) |
| Model 2 | 0.99 (0.97–1.00) | 1.00 (ref) | 0.70 (0.47–1.04) | 0.65 (0.44–0.96) * |
| **Prostate cancer** |  |  |  |  |
| No. of events/subjects | 89/6053 | 22/2050 | 32/1985 | 35/2018 |
| Univariate | 1.02 (1.00–1.04) | 1.00 (ref) | 1.50 (0.87–2.59) | 1.62 (0.95–2.77) |
| Model 1 | 1.00 (0.98–1.02) | 1.00 (ref) | 1.14 (0.66–1.96) | 0.95 (0.56–1.63) |
| Model 2 | 1.00 (0.97–1.02) | 1.00 (ref) | 1.16 (0.67–2.01) | 0.90 (0.52–1.56) |
| **Hematological cancer** |  |  |  |  |
| No. of events/subjects | 64/6053 | 28/2050 | 22/1985 | 14/2018 |
| Univariate | 0.97 (0.94–1.00) | 1.00 (ref) | 0.76 (0.43–1.34) | 0.49 (0.26–0.94) * |
| Model 1 | 0.96 (0.93–0.99) * | 1.00 (ref) | 0.67 (0.38–1.19) | 0.42 (0.22–0.80) * |
| Model 2 | 0.97 (0.94–1.00) * | 1.00 (ref) | 0.73 (0.41–1.30) | 0.45 (0.23–0.87) * |
| **Urothelial cell cancer** |  |  |  |  |
| No. of events/subjects | 78/6053 | 19/2050 | 33/1985 | 26/2018 |
| Univariate | 1.01 (0.99–1.04) | 1.00 (ref) | 1.80 (1.02–3.17) * | 1.39 (0.77–2.52) |
| Model 1 | 1.00 (0.97–1.02) | 1.00 (ref) | 1.48 (0.84–2.60) | 0.97 (0.53–1.76) |
| Model 2 | 1.00 (0.98–1.02) | 1.00 (ref) | 1.55 (0.87–2.76) | 1.03 (0.55–1.90) |

HRs and 95% CIs were derived from Cox proportional hazards regression models.
Model 1: adjusted for age, sex and race.
Model 2: as model 1+ adjusted for baseline eGFR, BMI, smoking, alcohol, educational level and type 2 diabetes, protein, diabetic medication, lipid lowering drugs, serum protein, hypertension, systolic blood pressure, UAE, hs-CRP and total cholesterol. Abbreviations: TSAT, transferrin saturation; sTfR, soluble transferrin receptor; BMI, body mass index; eGFR, estimated glomerular filtration rate; UAE, urinary albumin excretion; hs-CRP, high sensitivity c-reactive protein
*p* < 0.05: *; *p* < 0.01: **; *p* < 0.0625: ***

**Table S11|** Association of sTfR with cancer development for cancers diagnosed 1 year post blood sampling

|  | | | **Tertiles of sTfR (mg/L)** | | |
| --- | --- | --- | --- | --- | --- |
|  | **sTfR as**  **continuous variable** | **T1: < 2.2** | | **T2: 2.2-2.82** | **T3: >2.82** |
| **Overall cancer** |  |  | |  |  |
| No. of events/subjects | 608/6053 | 204/2018 | | 178/2044 | 226/1991 |
| Univariate | 1.26 (0.97–1.65) | 1.00 (ref) | | 0.96 (0.75–1.22) | 1.16 (0.95–1.42) |
| Model 1 | 1.15 (0.87–1.53) | 1.00 (ref) | | 0.86 (0.68–1.10) | 1.02 (0.84–1.25) |
| Model 2 | 1.31 (0.98–1.76) | 1.00 (ref) | | 0.94 (0.73–1.20) | 1.15 (0.93–1.42) |
| **Skin cancer** |  |  | |  |  |
| No. of events/subjects | 426/6053 | 120/2018 | | 158/2044 | 148/1991 |
| Univariate | 1.32 (0.96–1.82) | 1.00 (ref) | | 1.33 (1.04–1.69) * | 1.31 (1.02–1.68) * |
| Model 1 | 1.23 (0.87–1.75) | 1.00 (ref) | | 1.20 (0.95–1.53) | 1.16 (0.90–1.49) |
| Model 2 | 1.28 (0.89–1.84) | 1.00 (ref) | | 1.23 (0.96–1.57) | 1.20 (0.92–1.55) |
| **Lung cancer** |  |  | |  |  |
| No. of events/subjects | 101/6053 | 34/2018 | | 37/2044 | 30/1991 |
| Univariate | 1.02 (0.51–2.03) | 1.00 (ref) | | 1.37 (0.76–2.46) | 1.09 (0.61–1.97) |
| Model 1 | 0.76 (0.34–1.66) | 1.00 (ref) | | 1.14 (0.64–2.06) | 0.85 (0.47–1.56) |
| Model 2 | 1.09 (0.48–2.45) | 1.00 (ref) | | 1.37 (0.75–2.53) | 1.14 (0.61–2.15) |
| **Kidney cancer** |  |  | |  |  |
| No. of events/subjects | 95/6053 | 31/2018 | | 30/2044 | 34/1991 |
| Univariate | 1.14 (0.53–2.46) | 1.00 (ref) | | 0.98 (0.59–1.64) | 1.01 (0.58–1.77) |
| Model 1 | 0.89 (0.37–2.16) | 1.00 (ref) | | 0.83 (0.50–1.39) | 0.80 (0.46–1.39) |
| Model 2 | 1.04 (0.43–2.50) | 1.00 (ref) | | 0.92 (0.55–1.54) | 0.90 (0.51–1.60) |
| **Breast cancer** |  |  | |  |  |
| No. of events/subjects | 129/6053 | 44/2018 | | 43/2044 | 42/1991 |
| Univariate | 0.81 (0.45–1.45) | 1.00 (ref) | | 1.05 (0.66–1.65) | 0.98 (0.64–1.52) |
| Model 1 | 0.85 (0.49–1.50) | 1.00 (ref) | | 1.07 (0.68–1.69) | 1.02 (0.66–1.59) |
| Model 2 | 1.03 (0.57–1.85) | 1.00 (ref) | | 1.20 (0.76–1.91) | 1.21 (0.76–1.93) |
| **GI cancer** |  |  | |  |  |
| No. of events/subjects | 154/6053 | 63/2018 | | 42/2044 | 49/1991 |
| Univariate | 0.88 (0.51–1.50) | 1.00 (ref) | | 0.73 (0.47–1.13) | 0.85 (0.57–1.26) |
| Model 1 | 0.68 (0.38–1.25) | 1.00 (ref) | | 0.63 (0.41–0.98) * | 0.69 (0.46–1.04) |
| Model 2 | 0.88 (0.47–1.65) | 1.00 (ref) | | 0.71 (0.46–1.10) | 0.83 (0.54–1.28) |
| **Prostate cancer** |  |  | |  |  |
| No. of events/subjects | 89/6053 | 23/2018 | | 25/2044 | 41/1991 |
| Univariate | 2.07 (1.15–3.73) * | 1.00 (ref) | | 1.12 (0.60–2.10) | 1.89 (1.10–3.27) * |
| Model 1 | 1.69 (0.81–3.52) | 1.00 (ref) | | 0.89 (0.47–1.69) | 1.34 (0.77–2.34) |
| Model 2 | 1.62 (0.76–3.43) | 1.00 (ref) | | 0.83 (0.43–1.61) | 1.30 (0.73–2.34) |
| **Hematological cancer** |  |  | |  |  |
| No. of events/subjects | 64/6053 | 15/2018 | | 15/2044 | 34/1991 |
| Univariate | 1.98 (0.92–4.24) | 1.00 (ref) | | 1.22 (0.59–2.53) | 2.07 (1.09–3.93) * |
| Model 1 | 1.94 (0.84–4.45) | 1.00 (ref) | | 1.09 (0.53–2.26) | 1.80 (0.95–3.43) |
| Model 2 | 1.65 (0.69–3.95) | 1.00 (ref) | | 1.05 (0.51–2.19) | 1.69 (0.86–3.31) |
| **Urothelial cell cancer** |  |  | |  |  |
| No. of events/subjects | 78/6053 | 29/2018 | | 24/2044 | 25/1991 |
| Univariate | 0.78 (0.31–1.93) | 1.00 (ref) | | 0.83 (0.47–1.46) | 0.80 (0.43–1.50) |
| Model 1 | 0.51 (0.18–1.43) | 1.00 (ref) | | 0.68 (0.38–1.20) | 0.60 (0.32–1.11) |
| Model 2 | 0.61 (0.21–1.77) | 1.00 (ref) | | 0.74 (0.42–1.33) | 0.67 (0.35–1.28) |

HRs and 95% CIs were derived from Cox proportional hazards regression models.
Model 1: adjusted for age, sex and race.
Model 2: as model 1+ adjusted for baseline eGFR, BMI, smoking, alcohol, educational level and type 2 diabetes, protein, diabetic medication, lipid lowering drugs, serum protein, hypertension, systolic blood pressure, UAE, hs-CRP and total cholesterol. Abbreviations: sTfR, soluble transferrin receptor; BMI, body mass index; eGFR, estimated glomerular filtration rate; UAE, urinary albumin excretion; hs-CRP, high sensitivity c-reactive protein
*p* < 0.05: *; *p* < 0.01: **; *p* < 0.0625: ***

**Table S12|** Association of hepcidin with cancer development for cancers diagnosed 1 year post blood sampling

|  | | **Tertiles of hepcidin (nM)** | | |
| --- | --- | --- | --- | --- |
|  | **Hepcidin as**  **continuous variable** | **T1: < 2.1** | **T2: 2.1–4.1** | **T3: >4.1** |
| **Overall cancer** |  |  |  |  |
| No. of events/subjects | 608/6053 | 196/2017 | 179/2020 | 233/2016 |
| Univariate | 1.08 (0.99–1.18) | 1.00 (ref) | 0.92 (0.75–1.13) | 1.21 (1.00–1.46) |
| Model 1 | 0.88 (0.80–0.96) ** | 1.00 (ref) | 0.69 (0.56–0.85) *** | 0.79 (0.65–0.96) * |
| Model 2 | 0.87 (0.79–0.95) *** | 1.00 (ref) | 0.68 (0.56–0.84) *** | 0.77 (0.63–0.94) * |
| **Skin cancer** |  |  |  |  |
| No. of events/subjects | 426/6053 | 103/2017 | 158/2020 | 165/2016 |
| Univariate | 1.25 (1.12–1.40) *** | 1.00 (ref) | 1.57 (1.23–2.02) *** | 1.69 (1.32–2.16) *** |
| Model 1 | 1.03 (0.92–1.15) | 1.00 (ref) | 1.19 (0.92–1.53) | 1.12 (0.87–1.44) |
| Model 2 | 1.07 (0.95–1.20) | 1.00 (ref) | 1.24 (0.96–1.60) | 1.20 (0.92–1.56) |
| **Lung cancer** |  |  |  |  |
| No. of events/subjects | 101/6053 | 25/2017 | 26/2020 | 50/2016 |
| Univariate | 1.40 (1.11–1.77) *** | 1.00 (ref) | 1.09 (0.63–1.88) | 1.97 (1.21–3.21) ** |
| Model 1 | 1.07 (0.84–1.36) | 1.00 (ref) | 0.74 (0.43–1.28) | 1.10 (0.67–1.81) |
| Model 2 | 0.94 (0.74–1.21) | 1.00 (ref) | 0.65 (0.37–1.13) | 0.85 (0.51–1.44) |
| **Kidney cancer** |  |  |  |  |
| No. of events/subjects | 95/6053 | 20/2017 | 29/2020 | 46/2016 |
| Univariate | 1.54 (1.20–1.96) *** | 1.00 (ref) | 1.51 (0.84–2.69) | 2.43 (1.43–4.13) *** |
| Model 1 | 1.16 (0.90–1.50) | 1.00 (ref) | 0.98 (0.55–1.76) | 1.31 (0.77–2.25) |
| Model 2 | 1.15 (0.88–1.49) | 1.00 (ref) | 0.93 (0.52–1.69) | 1.28 (0.73–2.24) |
| **Breast cancer** |  |  |  |  |
| No. of events/subjects | 129/6053 | 58/2017 | 39/2020 | 32/2016 |
| Univariate | 0.71 (0.60–0.84) *** | 1.00 (ref) | 0.67 (0.45–1.01) | 0.56 (0.37–0.87) * |
| Model 1 | 0.84 (0.69–1.01) | 1.00 (ref) | 0.88 (0.57–1.34) | 0.83 (0.52–1.34) |
| Model 2 | 0.82 (0.67–1.00) | 1.00 (ref) | 0.87 (0.56–1.34) | 0.85 (0.52–1.38) |
| **GI cancer** |  |  |  |  |
| No. of events/subjects | 154/6053 | 47/2017 | 46/2020 | 61/2016 |
| Univariate | 1.11 (0.93–1.32) | 1.00 (ref) | 0.98 (0.65–1.47) | 1.34 (0.91–1.95) |
| Model 1 | 0.84 (0.70–1.00) | 1.00 (ref) | 0.66 (0.44–0.99) * | 0.75 (0.51–1.10) |
| Model 2 | 0.83 (0.69–1.01) | 1.00 (ref) | 0.67 (0.45–1.02) | 0.75 (0.51–1.12) |
| **Prostate cancer** |  |  |  |  |
| No. of events/subjects | 89/6053 | 23/2017 | 28/2020 | 38/2016 |
| Univariate | 1.34 (1.05–1.71) * | 1.00 (ref) | 1.15 (0.66–2.00) | 1.64 (0.98–2.74) |
| Model 1 | 0.91 (0.71–1.16) | 1.00 (ref) | 0.67 (0.38–1.17) | 0.72 (0.43–1.21) |
| Model 2 | 0.93 (0.72–1.21) | 1.00 (ref) | 0.71 (0.40–1.26) | 0.79 (0.46–1.36) |
| **Hematological cancer** |  |  |  |  |
| No. of events/subjects | 64/6053 | 21/2017 | 13/2020 | 30/2016 |
| Univariate | 1.32 (0.99–1.77) | 1.00 (ref) | 0.62 (0.31–1.24) | 1.47 (0.84–2.58) |
| Model 1 | 1.07 (0.80–1.45) | 1.00 (ref) | 0.46 (0.23–0.92) * | 0.93 (0.53–1.66) |
| Model 2 | 1.00 (0.74–1.36) | 1.00 (ref) | 0.46 (0.23–0.94) * | 0.85 (0.46–1.56) |
| **Urothelial cell cancer** |  |  |  |  |
| No. of events/subjects | 78/6053 | 13/2017 | 27/2020 | 38/2016 |
| Univariate | 1.71 (1.30–2.25) *** | 1.00 (ref) | 2.09 (1.08–4.05) * | 3.01 (1.61–5.66) *** |
| Model 1 | 1.28 (0.96–1.71) | 1.00 (ref) | 1.33 (0.69–2.58) | 1.57 (0.83–2.95) |
| Model 2 | 1.27 (0.94–1.71) | 1.00 (ref) | 1.27 (0.65–2.49) | 1.52 (0.79–2.92) |

HRs and 95% CIs were derived from Cox proportional hazards regression models.
Model 1: adjusted for age, sex and race.
Model 2: as model 1+ adjusted for baseline eGFR, BMI, smoking, alcohol, educational level and type 2 diabetes, protein, diabetic medication, lipid lowering drugs, serum protein, hypertension, systolic blood pressure, UAE, hs-CRP and total cholesterol. Abbreviations: BMI, body mass index; eGFR, estimated glomerular filtration rate; UAE, urinary albumin excretion; hs-CRP, high sensitivity c-reactive protein
*p* < 0.05: *; *p* < 0.01: **; *p* < 0.0625: ***

**Table S13|** Association of EPO with cancer development for cancers diagnosed 1 year post blood sampling

|  | | Tertiles of EPO (IU/L) | | |
| --- | --- | --- | --- | --- |
|  | **EPO as**  **continuous variable** | **T1: < 6.50** | **T2: 6.50-9.22** | **T3: >9.22** |
| Overall cancer |  |  |  |  |
| No. of events/subjects | 608/6053 | 172/2030 | 206/2011 | 230/2012 |
| Univariate | 1.32 (1.13–1.54) *** | 1.00 (ref) | 1.22 (1.00–1.50) | 1.40 (1.14–1.71) *** |
| Model 1 | 1.25 (1.06–1.47) ** | 1.00 (ref) | 1.16 (0.95–1.42) | 1.22 (1.00–1.50) |
| Model 2 | 1.23 (1.04–1.44) * | 1.00 (ref) | 1.16 (0.95–1.43) | 1.21 (0.98–1.49) |
| Skin cancer |  |  |  |  |
| No. of events/subjects | 426/6053 | 132/2030 | 146/2011 | 148/2012 |
| Univariate | 1.01 (0.83–1.23) | 1.00 (ref) | 1.12 (0.88–1.41) | 1.13 (0.89–1.43) |
| Model 1 | 0.94 (0.77–1.14) | 1.00 (ref) | 1.07 (0.84–1.35) | 0.98 (0.77–1.24) |
| Model 2 | 0.91 (0.74–1.12) | 1.00 (ref) | 1.06 (0.84–1.35) | 0.95 (0.75–1.22) |
| Lung cancer |  |  |  |  |
| No. of events/subjects | 101/6053 | 35/2030 | 30/2011 | 36/2012 |
| Univariate | 1.00 (0.67–1.49) | 1.00 (ref) | 0.89 (0.55–1.46) | 1.07 (0.67–1.72) |
| Model 1 | 0.88 (0.58–1.33) | 1.00 (ref) | 0.83 (0.51–1.36) | 0.88 (0.54–1.41) |
| Model 2 | 0.99 (0.65–1.52) | 1.00 (ref) | 0.88 (0.53–1.45) | 0.96 (0.58–1.58) |
| Kidney cancer |  |  |  |  |
| No. of events/subjects | 95/6053 | 25/2030 | 39/2011 | 31/2012 |
| Univariate | 1.22 (0.82–1.80) | 1.00 (ref) | 1.62 (0.98–2.70) | 1.32 (0.77–2.25) |
| Model 1 | 1.15 (0.75–1.76) | 1.00 (ref) | 1.56 (0.94–2.59) | 1.12 (0.65–1.92) |
| Model 2 | 1.08 (0.70–1.68) | 1.00 (ref) | 1.55 (0.93–2.60) | 1.05 (0.60–1.83) |
| Breast cancer |  |  |  |  |
| No. of events/subjects | 129/6053 | 45/2030 | 44/2011 | 40/2012 |
| Univariate | 0.99 (0.70–1.41) | 1.00 (ref) | 1.00 (0.66–1.53) | 0.89 (0.58–1.37) |
| Model 1 | 0.85 (0.60–1.20) | 1.00 (ref) | 0.93 (0.61–1.42) | 0.74 (0.48–1.14) |
| Model 2 | 0.84 (0.59–1.20) | 1.00 (ref) | 0.91 (0.6–1.39) | 0.73 (0.46–1.13) |
| GI cancer |  |  |  |  |
| No. of events/subjects | 154/6053 | 48/2030 | 47/2011 | 59/2012 |
| Univariate | 1.15 (0.83–1.58) | 1.00 (ref) | 1.01 (0.68–1.52) | 1.27 (0.87–1.87) |
| Model 1 | 1.07 (0.76–1.51) | 1.00 (ref) | 0.96 (0.64–1.44) | 1.09 (0.74–1.60) |
| Model 2 | 1.13 (0.80–1.60) | 1.00 (ref) | 1.01 (0.67–1.51) | 1.18 (0.79–1.77) |
| Prostate cancer |  |  |  |  |
| No. of events/subjects | 89/6053 | 21/2030 | 32/2011 | 36/2012 |
| Univariate | 1.52 (1.05–2.2) *** | 1.00 (ref) | 1.59 (0.91–2.78) | 1.83 (1.06–3.18) * |
| Model 1 | 1.52 (1–2.31) *** | 1.00 (ref) | 1.53 (0.88–2.68) | 1.57 (0.90–2.73) |
| Model 2 | 1.44 (0.94–2.22) | 1.00 (ref) | 1.5 (0.85–2.64) | 1.52 (0.86–2.70) |
| Hematological cancer |  |  |  |  |
| No. of events/subjects | 64/6053 | 17/2030 | 24/2011 | 23/2012 |
| Univariate | 1.42 (0.90–2.22) | 1.00 (ref) | 1.38 (0.74–2.59) | 1.39 (0.74–2.60) |
| Model 1 | 1.34 (0.82–2.20) | 1.00 (ref) | 1.30 (0.69–2.44) | 1.17 (0.62–2.21) |
| Model 2 | 1.18 (0.71–1.96) | 1.00 (ref) | 1.29 (0.68–2.43) | 1.00 (0.52–1.93) |
| Urothelial cell cancer |  |  |  |  |
| No. of events/subjects | 78/6053 | 23/2030 | 30/2011 | 25/2012 |
| Univariate | 1.01 (0.64–1.59) | 1.00 (ref) | 1.36 (0.78–2.35) | 1.16 (0.65–2.07) |
| Model 1 | 0.90 (0.56–1.44) | 1.00 (ref) | 1.29 (0.74–2.23) | 0.94 (0.53–1.69) |
| Model 2 | 0.90 (0.55–1.47) | 1.00 (ref) | 1.33 (0.76–2.32) | 0.95 (0.52–1.74) |

HRs and 95% CIs were derived from Cox proportional hazards regression models.
Model 1: adjusted for age, sex and race.
Model 2: as model 1+ adjusted for baseline eGFR, BMI, smoking, alcohol, educational level and type 2 diabetes, protein, diabetic medication, lipid lowering drugs, serum protein, hypertension, systolic blood pressure, UAE, hs-CRP and total cholesterol. Abbreviations: EPO, erythropoietin; BMI, body mass index; eGFR, estimated glomerular filtration rate; UAE, urinary albumin excretion; hs-CRP, high sensitivity c-reactive protein
*p* < 0.05: *; *p* < 0.01: **; *p* < 0.0625: ***

|  | Continuous variable | Tertiles of the respective iron parameters or EPO levels | | |
| --- | --- | --- | --- | --- |
| Association of sTfR with cancer development for cancers in non-smokers | | | | |
|  |  | **T1: < 2.3 mg/L** | **T2: 2.3-2.8 mg/L** | **T3: >2.8 mg/L** |
| No. of events/subjects | 645/6109 | 202/2043 | 205/2032 | 238/2034 |
| Model 2 | 1.68 (1.21–2.32) *** | 1.00 (ref) | 1.00 (0.75–1.34) | 1.24 (0.94–1.64) |
| Association of sTfR with cancer development for cancers in smokers | | | | |
|  |  | **T1: < 2.1 mg/L** | **T2: 2.1-2.6 mg/L** | **T3: > 2.6 mg/L** |
| No. of events/subjects | 645/6109 | 202/2043 | 205/2032 | 238/2034 |
| Model 2 | 0.81 (0.47–1.39) | 1.00 (ref) | 0.87 (0.61–1.25) | 0.89 (0.58–1.36) |
| Association of hepcidin with cancer development for cancers in individuals with a low BMI | | | | |
|  |  | **T1: < 1.6 nM** | **T2: 1.6-3.3 nM** | **T3: >3.3 nM** |
| No. of events/subjects | 645/6109 | 208/2037 | 191/2033 | 246/2039 |
| Model 2 | 0.77 (0.66–0.90) *** | 1.00 (ref) | 0.57 (0.41–0.79) *** | 0.56 (0.39–0.81) *** |
| Association of hepcidin with cancer development for cancers in individuals with a high BMI | | | | |
|  |  | **T1: < 2.5 nM** | **T2: 1.5-3.4 nM** | **T3: > 3.4 nM** |
| No. of events/subjects | 645/6109 | 208/2037 | 191/2033 | 246/2039 |
| Model 2 | 0.95 (0.85–1.07) | 1.00 (ref) | 0.79 (0.61–1.03) | 0.93 (0.73–1.20) |
| Association of hepcidin with cancer development for cancers in male | | | | |
|  |  | **T: <2.7 nM** | **T2: 2.7-4.7 nM** | **T3: >4.7** |
| No. of events/subjects | 645/6109 | 208/2037 | 191/2033 | 246/2039 |
| Model 2 | 0.96 (0.84-1.09) | 1.00 (ref) | 0.55 (0.55-0.98)* | 0.86 (0.66-1.13) |
| Association of hepcidin with cancer development for cancers in female | | | | |
|  |  | **T1: < 1.5** | **T2: 1.5-3.4 nM** | **T3: >3.4** |
| No. of events/subjects | 645/6109 | 208/2037 | 191/2033 | 246/2039 |
| Model 2 | 0.89 (0.82–0.98) * | 1.00 (ref) | 0.74 (0.55-0.99) * | 0.79 (0.57-1.08) |
| Association of EPO with cancer development for cancers in non-smokers | | | | |
|  |  | **T1: < 6.6 IU/L** | **T2: 6.6-9.4** | **T3: > 9.4** |
| No. of events/subjects | 645/6109 | 176/2038 | 224/2027 | 245/2044 |
| Model 2 | 1.40 (1.14–1.72) *** | 1.00 (ref) | 1.47 (1.13–1.92) *** | 1.42 (1.09–1.84) ** |
| Association of EPO with cancer development for cancers in smokers | | | | |
|  |  | **T1: <6.1 IU/L** | **T2: 6.1-8.6** | **T3: > 8.6** |
| No. of events/subjects | 645/6109 | 176/2038 | 224/2027 | 245/2044 |
| Model 2 | 1.01 (0.75–1.34) | 1.00 (ref) | 0.92 (0.66–1.27) | 0.96 (0.68–1.35) |

**Table S14|** Association of iron parameters and EPO levels with significant effect modifications with cancer development over time

HRs and 95% CIs were derived from Cox proportional hazards regression models.
Model 2: Adjusted for sex, age, race, baseline eGFR, BMI, smoking, alcohol, educational level and type 2 diabetes, protein, diabetic medication, lipid lowering drugs, serum protein, hypertension, systolic blood pressure, UAE, hs-CRP and total cholesterol.

Abbreviations: sTfR, soluble transferrin receptor; EPO, erythropoietin; BMI, body mass index; eGFR, estimated glomerular filtration rate; UAE, urinary albumin excretion; hs-CRP, high sensitivity c-reactive protein

*p* < 0.05: *; *p* < 0.01: **; *p* < 0.0625: ***

**Figure S1:**  Forest plot showing effect modification by different subgroups on the cancer incidence for TSAT, sTfR and hepcidin.

Subgroup analyses investigating effect modification of the association TSAT, sTfR, and hepcidin with cancer incidence by age, sex, smoking status, BMI, and eGFR. HRs and 95% CIs were derived from Cox proportional hazards regression models. HR were adjusted for age, sex, BMI, smoking, alcohol, educational level, type 2 diabetes, and baseline eGFR. Abbreviations: BMI, body mass index; CI, confidence interval; eGFR, estimated glomerular filtration rate; EPO, erythropoietin; sTfR, soluble transferrin receptor; TSAT, transferrin saturation.
